# Chromosome-scale assembly and population diversity analyses provide insights into the evolution of *Sapindus mukorossi*

**DOI:** 10.1093/hr/uhac012

**Published:** 2022-02-17

**Authors:** Ting Xue, Duo Chen, Tianyu Zhang, Youqiang Chen, Huihua Fan, Yunpeng Huang, Quanlin Zhong, Baoyin Li

**Affiliations:** Fujian Provincial Key Laboratory for Plant Eco-physiology, State Key Laboratory for Subtropical Mountain Ecology of the Ministry of Science and Technology and Fujian Province, College of Geographical Sciences, Fujian Normal University, Fuzhou 350007, China; College of Life Sciences, Fujian Normal University, Fuzhou 350117, China; College of Life Sciences, Fujian Normal University, Fuzhou 350117, China; Shunchang County Forestry Science and Technology Center of Fujian Province, Forestry Bureau of Shunchang, Shunchang 353200, China; College of Life Sciences, Fujian Normal University, Fuzhou 350117, China; Research Institute of Forestry, Fujian Research Institute of Forestry, Fuzhou 350000, China; Research Institute of Forestry, Fujian Research Institute of Forestry, Fuzhou 350000, China; Fujian Provincial Key Laboratory for Plant Eco-physiology, State Key Laboratory for Subtropical Mountain Ecology of the Ministry of Science and Technology and Fujian Province, College of Geographical Sciences, Fujian Normal University, Fuzhou 350007, China; Fujian Provincial Key Laboratory for Plant Eco-physiology, State Key Laboratory for Subtropical Mountain Ecology of the Ministry of Science and Technology and Fujian Province, College of Geographical Sciences, Fujian Normal University, Fuzhou 350007, China

**Keywords:** Horticultural plant genomes II

## Abstract

*Sapindus mukorossi* is an environmentally friendly plant and renewable energy source whose fruit has been widely used for biomedicine, biodiesel, and biological chemicals due to its richness in saponin and oil contents. Here, we report the first chromosome-scale genome assembly of *S. mukorossi* (covering ~391 Mb with a scaffold N50 of 24.66 Mb) and characterize its genetic architecture and evolution by resequencing 104 *S. mukorossi* accessions. Population genetic analyses showed that genetic diversity in the southwestern distribution area was relatively higher than that in the northeastern distribution area. Gene flow events indicated that southwest species may be the donor population for the distribution areas in China. Genome-wide selective sweep analysis showed that a large number of genes are involved in defense responses, growth and development, including *SmRPS2*, *SmRPS4*, *SmRPS7*, *SmNAC2*, *SmNAC23*, *SmNAC102*, *SmWRKY6*, *SmWRKY26*, and *SmWRKY33.* We also identified several candidate genes controlling six agronomic traits by genome-wide association studies, including *SmPCBP2*, *SmbHLH1*, *SmCSLD1*, *SmPP2C*, *SmLRR-RKs*, and *SmAHP.* Our study not only provides a rich genomic resource for further basic research on Sapindaceae woody trees but also identifies several economically significant genes for genomics-enabled improvements in molecular breeding.

## Teaser

We report the first chromosome-scale genome assembly of *Sapindus mukorossi*, covering ~391 Mb with a scaffold N50 of 24.66 Mb.

Population genetic analyses showed that genetic diversity in the southwest of the distribution area is relatively higher than that in the northeast.

Genome-wide selective sweep analysis showed that the selection of a large number of genes is involved in defense responses, growth, and development.

Our identified candidate genes are associated with major agronomic traits, and this information can be used for further reference in the selection of superior germplasm resources.

## Introduction

**Figure 1:**
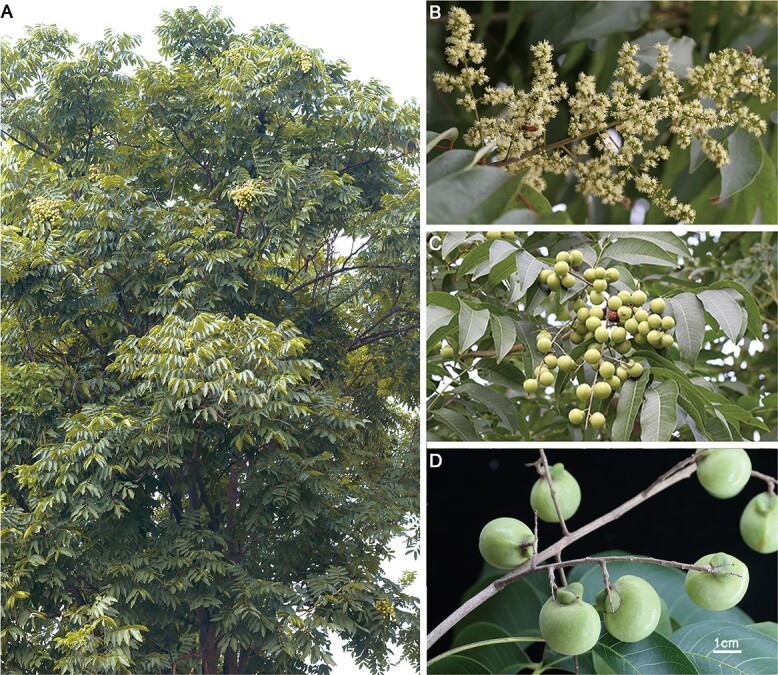
Representative photographs of *S. mukorossi*. **a** Mature *S. mukorossi* tree in its natural habitat. **b** Flowers. **c, d** Fruits. Bar =1 cm.

*Sapindus mukorossi* (Fig. 1), also known as soapberry tree, is a deciduous tree of the Sapindaceae family and is widely distributed in Southeast Asia, Japan, and southern and southwestern China [1]. Li Shizhen wrote ‘By using *S. mukorossi*, shampoo can go to head wind, and washing can whiten’ in *The Compendium of Materia Medica*, highlighting that *S. mukorossi* is a natural excellent detergent with good foaming and decontamination abilities [2]. As recorded in *Pu Ji Fang*, ‘*S. mukorossi* can also treat toothache, sore throat, acute gastroenteritis and so on’, showing that *S. mukorossi* is a very important Chinese herbal medicine with multiple biomedical functions [3]*.* The fruit peel of *S. mukorossi* is rich in saponins (up to 10.76%) with physiological activities, such as antifungal, antidandruff and anti-itching activities [4, 5]. The seeds of *S. mukorossi* are rich in oil (up to 42.70%) with a high proportion of unsaturated fatty acids (up to 86.63%), rendering them ideal materials for developing biodiesel and coping with the energy crisis [6, 7]. With its richness in saponins in the peel and oil content in kernels, *S. mukorossi* has become an important industrial tree species that can provide multifunctional raw material for producing biological chemicals, biodiesel, and biomedicine [8, 9]. Additionally, with a beautiful crown shape, strong adaptability, and resistance to sulfur dioxide and industrial dust pollution, *S. mukorossi* is regarded as a new type of low-carbon, environmentally friendly renewable energy source for ecological restoration of barren mountains and rural greening [10, 11].

Based on a large number of studies, research on *S. mukorossi* mainly focuses on genetic and ecological characteristics, seed germination, landscaping, active ingredient extraction, etc. [12–16]. For example, Liu *et al*. comprehensively evaluated superior germplasms from natural germplasm resources of three species and one variety of *Sapindus* in China and Vietnam and divided them into three categories, each with 10 selected superior germplasm accessions for oil and saponins [17]. Phenotypic and molecular markers (inter simple sequence repeat, simple sequence repeat and random amplified polymorphic DNA) were used to analyze the diversity of natural germplasm resources in India and China in different geographical regions. The results showed that the variation between individuals within populations is higher than that between populations [18–22]. Fan *et al*. investigated 122 accessions from nine provinces by measuring the main economic traits (saponins, oil, age, height, altitude, etc.), which showed that various economic traits of trees of different origins have obvious differences, and the main economic traits of different trees from the same area are also different [11]. However, previous studies have focused on small samples, resulting in only partial representation of *S. mukorossi* based on fruit and seed traits, and no studies have explored the related omics and molecular breeding of *S. mukorossi*. The abundance of germplasm resources is a prerequisite for the selection of improved varieties, and the genetic diversity of germplasm resources is the material basis for biological evolution and breeding [23]. Population resequencing has become an important strategy for further crop domestication, improvement, and breeding, as recently reported in jujube [24], soybean [25], chickpea [26], and peach [27]. Population resequencing studies have been performed to examine the genetic diversity and genetic basis of evolution and adaptation in species such as *Salix brachista* [28] and tea [29]. In theory, different species have experienced reproductive or geographical isolation. Due to the long evolutionary history of *S. mukorossi*, after long-term natural selection various morphological variations and biological genetic diversity in geographical areas are objectively present in nature. Moreover, different *S. mukorossi* varieties are easily cultivated by hybridization, complicating accurate tracing of the origin of the offspring. Although previous studies have attempted to explore this issue, the lack of genome data and annotated gene sets for *S. mukorossi* has limited functional studies and evolutionary analyses.

To gain a better understanding of the origin and evolution of *S. mukorossi*, we used PacBio and Hi-C technologies to assemble a genome, providing a good resource for investigating genetic diversity and evolution. We also collected 104 *S. mukorossi* resources for resequencing from nine provinces of China covering nearly all *S. mukorossi* planting areas at the whole-genome level, including 47 accessions from Northeast China (Anhui Province, AH; Jiangxi Province, JX; Fujian Province, FJ; Zhejiang Province, ZJ), 38 accessions from South China (Hubei Province, HB; Hunan Province, HN; FJ, JX, Guangdong Province, GD; Guangxi Province, GX), and 18 accessions from Southwest China (Yunnan Province, YN) (Supplementary Table S1). Using these sequencing data, we demonstrated the evolutionary history of *S. mukorossi* and revealed the phylogenetic relationship between these populations by analyzing genomic characteristics, population structures, and genetic diversity. To identify potential candidate genes associated with fruit weight, peel-to-fruit ratio, saponin contents, seed-to-fruit ratio, kernel-to-fruit ratio, and oil contents, we also performed genome-wide association studies (GWAS) using resequencing data from 57 accessions (Supplementary Table S2). Our results provide a new resource for further molecular breeding and studies of *S. mukorossi* biology. However, genome-wide association analyses of economic traits (saponins, oil, etc.) remain to be conducted in the future.

## Results

### Sequencing and assembly of the *S. mukorossi* genome

**Figure 2:**
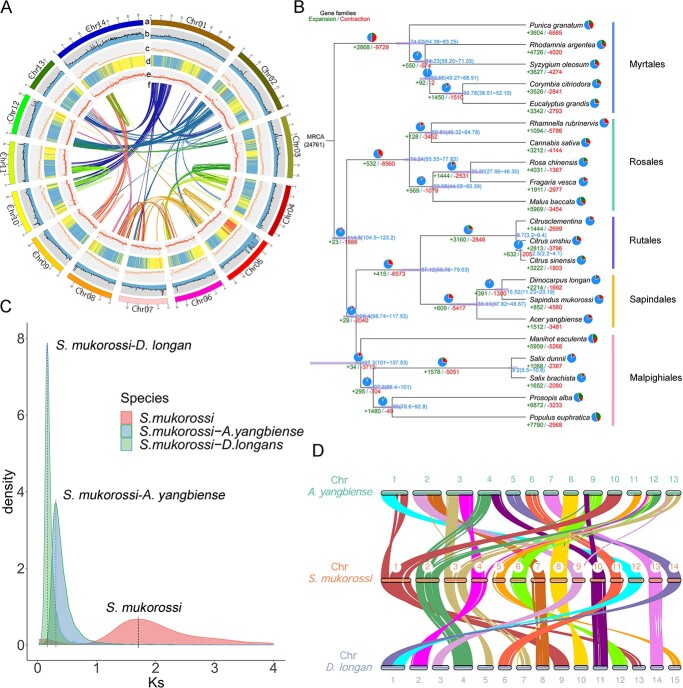
Characterization and evolution of the *S. mukorossi* genome. **a** Genome features across 14 chromosomes. (a) The 14 assembled chromosomes; (b) GC content; (c) gene density; (d) summary of gene expression in roots, stems, and leaves; (e) Transposable element density over the genome in non-overlapping windows; (f) summary of major interchromosomal syntenic relationships. Central colored lines represent syntenic blocks; block size = 3 kb. **b** Phylogenetic tree and evolution analysis of *S. mukorossi*. Red and green fonts indicate the numbers of gene families that have expanded and contracted among 21 species, respectively. Gene family expansions are indicated in green in the pie chart, while gene family contractions are indicated in red. Bars represent the 95% highest probability densities of the estimated divergence time. **c***K*s distribution of syntenic blocks between *S. mukorossi*, *D. longan*, and *A. yangbiense*. **d** Macrosynteny analysis between *S. mukorossi*, *D. longan*, and *A. yangbiense*. Each line connects a pair of orthologous genes.

Approximately 21 Gb (~47×) of Illumina paired-end sequences were generated and used to estimate genome size and heterozygosity using Jellyfish software, which indicated that the size of the *S. mukorossi* genome was 432.29 Mb with 1.52% heterozygosity (Supplementary Fig. S1; Supplementary Table S3). A total of 22.96 Gb of HiFi circular consensus sequencing (CCS) data were obtained, yielding a *de novo* contig-level assembly of 391.55 Mb with a GC content of 33.20% and a contig N50 of 2.88 Mb, which was close to the size estimated according to flow cytometry (Fig. 2a; Supplementary Fig. S2; Supplementary Table S4) and genome survey analyses (Supplementary Fig. S1). The assembled genome size was smaller than those of other closely related species in Sapindales, such as *Dimocarpus longan* (~471 Mb) and *Acer yangbiense* (~666 Mb) [30, 31]. Regarding the chromosome number, *S. mukorossi* is a diploid species, which has a karyotype of 2*n* = 2*x* = 28 homologous pairs of chromosomes based on karyotyping analysis (Supplementary Fig. S3). To obtain a chromosome-scale assembly of *S. mukorossi*, a total of 40.96 Gb of clean sequences were generated by Hi-C sequencing. Using the contact matrix and the agglomerative hierarchical clustering method in LACHESIS, 90.87% (~352.74 Mb) of the assembled genome was successfully anchored into 14 pseudochromosomes (the length of the pseudochromosomes ranged from 15.76 to 44.80 Mb) with a scaffold N50 value of 24.66 Mb, which is 8.56-fold higher than that of the *de novo* assembled genome (a contig N50 of 2.88 Mb) (Fig. 2a; Supplementary Fig. S4; Supplementary Table S5). Expressed sequence tags (EST; ~94.09% of transcriptome data) and Benchmarking Universal Single-Copy Orthologs (BUSCO; ~83.80% of complete BUSCOs) analyses showed high accuracy and good completeness of the assembly (Supplementary Tables S6 and S7).

### Gene prediction and annotation

A total of 31 853 protein-coding genes were identified in the *S. mukorossi* genome, which is higher than the numbers in other closely related species, such as *D. longan* (31  007 proteins) and *A. yangbiense* (28 320 proteins). The average gene length was 2978 bp and the average coding sequence (CDS) length was 1524 bp. Among the 31 853 predicted proteins, 27 509 (86.36%) were functionally annotated from five known databases: NR (27 335 genes, 85.82%), Swiss-Prot (23 766 genes, 74.61%), GO (Gene Ontology; 18 615 genes, 58.44%), KOG (Eukaryotic Orthologous Groups of Proteins; 14 452 genes, 45.37%), and KEGG (Kyoto Encyclopedia of Genes and Genomes; 11 025 genes, 34.61%) (Supplementary Fig. S5; Supplementary Table S8). We identified 2140 transcription factors (TFs) distributed in 35 families, representing 6.72% of genes in the *S. mukorossi* genome, which is ~1.33-fold higher than that in the *D. longan* genome (1611, 4.1%) (Supplementary Table S9). The identification of these TFs in the *S. mukorossi* genome will help elucidate major secondary metabolite biosynthesis in the future. We further identified a total length of 57.16 Mb of repetitive elements, representing 14.60% of the assembled genome (Supplementary Table S10), which is significantly lower than the amount reported in *D. longan* (52.87%, 261.88 Mb), indicating that the sizes of the *S. mukorossi* and *D. longan* genomes are different due to the number of repetitive elements that they contain. Various fruit tree genomes have been reported to contain relatively high percentages of repetitive elements, including *D. longan* (52.87%), kiwifruit (36%), pineapple (38.3%), *Vitis vinifera* (41.4%), *Ziziphus jujuba* (49.49%), *Citrus sinensis* (51.9%), pear (53.1%), and apple (67.4%) [30, 32–38]. We speculated that these large numbers of repetitive sequences are specific to fruit species, which may be an important reason for the edibility of their pulp. Among the identified repetitive elements, 8.91% (34 883 414 bp) were long terminal repeats (LTRs) and 0.36% (1 402 605 bp) were long interspersed nuclear elements (LINEs) (Supplementary Table S10). In addition, 4018 *C. sinensis* tRNAs, 88 miRNAs, 3035 rRNAs, 572 snRNAs, 2675 introns, and 1 sRNA were detected (Supplementary Table S11).

### Genome evolution

Evolutionary analysis was performed using OrthoFinder (version 2.3.12) among 23 species representing five orders, including Myrtales, Rosales, Malpighiales, Rutales, and Sapindales. The phylogenetic tree showed that *S. mukorossi* had a closer relationship to *D. longan* and *A. yangbiense* and phylogenetically diverged from the common ancestor (Sapindales) (Fig. 2b). We identified 24 761 gene families and analyzed their evolution by comparing 21 plant species, and 852 expanded and 4580 contracted gene families were detected in the *S. mukorossi* genome. Interestingly, we found that the numbers of contracted and expanded gene families in *S. mukorossi* were 2.71- and 0.56-fold those in *D. longan* (1692 contracted and 852 expanded gene families), respectively. Enrichment analysis of contracted and expanded gene families was carried out with the following thresholds: *P* < .05 and false discovery rate < 0.05. GO and KEGG enrichment results showed that the expanded gene families of *S. mukorossi* were mostly enriched in cell, cell part, cellular process, energy metabolism, carbohydrate metabolism, cell growth, and lipid metabolism (Supplementary Tables S12 and –). These families included ATP-dependent Clp protease (*SmCLPP*, whz_003864), which plays an important role in the cell cycle and development; acetyl-CoA carboxylase carboxyl transferase (*SmAccD*, whz_015069), and alcohol dehydrogenase I (*SmADH1*, whz_020025), which are associated with cell envelope lipid biosynthesis and α-linolenic acid metabolism; and steroid 17α-monooxygenase (*SmCYP17A*, whz_000520), which participates in the synthesis of steroids, fatty acids, and eicosanoids. KEGG enrichment results showed that these contracted gene families were mostly enriched in starch and sucrose metabolism, hormone signal transduction, and sesquiterpenoid and triterpenoid biosynthesis (Supplementary Table ). These families included trehalose 6-phosphate synthase (*SmTPS1*, whz_009200), β-glucosidase (whz_002676), fructokinase (*SmFK*, whz_000626), sucrose-phosphate synthase (*SmSPS*, whz_024199), glucan endo-1,3-β-glucosidase 5 (whz_002416), and sucrose synthase (*SmSUS1*, whz_023880), some of which are involved in the biosynthesis of polysaccharides. Furthermore, we also identified 9521 gene families specific to the *S. mukorossi* genome among the nine plants. KEGG enrichment analysis showed that these gene families mainly participated in energy metabolism, environmental adaptation, carbohydrate metabolism, cell growth, signal transduction, and lipid metabolism (Supplementary Table ). These families included *SmRPM1* (whz_000421), *SmRPS2* (whz_000391) and *SmCML* (whz_000614), which are associated with disease resistance via plant–pathogen interactions; 3-oxoacyl-[acyl-carrier protein] reductase (whz_003695), enoyl-[acyl-carrier protein] reductase I (whz_001582), acetyl-CoA acyltransferase 1 (whz_005588), lipoxygenase (whz_001602), and linoleate 9S-lipoxygenase (whz_003125), which participate in linoleic acid metabolism and α-linolenic acid metabolism; and mevalonate kinase (*SmMVK*, whz_024682), mevalonate pyrophosphate decarboxylase (*SmMVD*, whz_005809), and squalene synthase (*SmSS*, whz_013402), which are involved in the hederagenin biosynthetic pathway. A total of 126 candidate genes in *S. mukorossi* appeared to be under positive selection according to the following parameters: *K*a/*K*s > 1, *P* < .05. Most of these genes were enriched in GO terms and KEGG pathways related to energy metabolism, cell growth and death, and stimulus response, including *Sm*ND5 (whz_000010), *Sm*ND2 (whz_006058), *Sm*CDC4 (whz_004052), and *Sm*NPR1 (whz_008089). We also found that among these positively selected TF families, *Sm*ARR-B (cytokinin signaling, whz_000208) and *Sm*B3 [the auxin response factor (ARF) family and the LEAFY COTYLEDON 2-ABSCISIC ACID INSENSITIVE 3-VP1/ABI3-LIKE (LAV) family, whz_004052] were involved in hormone signaling pathways during seed development (Supplementary Table S16).

To estimate the nature of whole-genome duplication (WGD) events in *S. mukorossi*, the *K*s distribution of homologous genes from *S. mukorossi*, *D. longan*, and *A. yangbiense* was characterized*.* Two *K*s values peaked at 0.18 and 0.36 for orthologs between *S. mukorossi* and *D. longan* and between *S. mukorossi* and *A. yangbiense*, suggesting speciation events at ~15 and ~31 Mya, which is consistent with the findings of the phylogenetic tree. The major peak of duplication in *S. mukorossi* was 1.90, which indicates that an ancient WGD event had occurred at ~161 Mya but did not reveal a recent WGD event, which is consistent with the *K*s peaks reported for *D. longan* and *A. yangbiense* (Fig. 2c)*.* These results indicated that the ancient WGD event of *S*. *mukorossi* occurred before the divergence of *S. mukorossi* and *D. longan*. Although synteny block analysis revealed strong collinearity in *S. mukorossi*, *D. longan*, and *A. yangbiense*, large interchromosomal rearrangements were detected for the homologous chromosome pairs AyChr03/SmChr04, SmChr04/DlChr02, AyChr08/SmChr08, AyChr01/SmChr01, SmChr02/DlChr04, SmChr03/DlChr05, SmChr07/DlChr08, and SmChr13/DlChr14 (Fig. 2d). In addition, partial chromosomal fusions were also observed in the homologous chromosome pairs among these species (AyChr01/Chr10 →SmChr01, AyChr03/Chr13 → SmChr03, AyChr04/Chr12 → SmChr02, DlChr09/Chr13 → SmChr01, SmChr12/Chr14 → DlChr01, DlChr05/Chr07 → SmChr03) despite high collinearity (Fig. 2d). These results suggested that major chromosomal fusions and rearrangements may have led to the divergence of the three species in Sapindaceae, which might be one of the reasons for the difference in chromosome formation.

**Figure 3:**
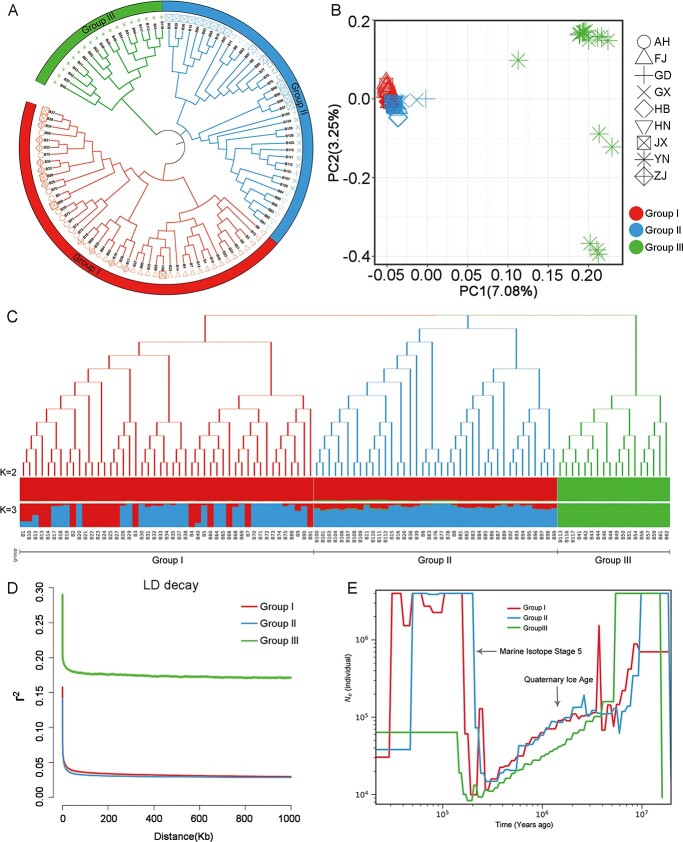
Phylogeny and population genetics of 104 *S. mukorossi* accessions. **a** Phylogenetic tree of 10 *S. mukorossi* accessions constructed based on whole-genome SNPs. **b** PCA plots of 104 *S. mukorossi* accessions. Different colors and shapes represent different subgroups and regions, respectively. **c** Population structure analysis of 104 *S. mukorossi* accessions (*K* = 2 or 3). The background of each putative ancestor is shown in different colors. **d** Genome-wide decay of LD. **e** Demographic history of *N*_e_ with a generation time of 5 years. Neutral mutation rate per generation (*μ*) = 1.25 × 10^−8^.

### Population genetic analysis

A total of 104 *S. mukorossi* accessions were collected from nine provinces of China covering nearly all *S. mukorossi* planting areas (Supplementary Table S1). Resequencing of the 104 *S. mukorossi* accessions by Illumina HiSeq PE150 generated a total of 545.83 Gb of clean data with an average depth of 12.27× per or 4.91 Gb of clean data per sample (Supplementary Table S3). After mapping against the assembled reference genome, we identified a total of 19 443 452 high-quality single-nucleotide polymorphisms (SNPs) and 7 960 863 InDels after filtering. Phylogenetic tree and principal component analysis (PCA) revealed that 104 samples were clustered into three independent groups corresponding to the Group I, Group II, and Group III populations (Fig. 3a and b). Group I mainly consisted of accessions from the northeast distribution area, with enrichment of accessions from FJ and ZJ. Group II mainly consisted of accessions from the southern distribution area, with enrichment of accessions from GX and JX. The accessions of the southwestern distribution area (YN) were placed in an independent branch as Group III, showing stronger geographical distribution patterns than the other two groups. To further analyze the genetic relationship between these accessions, we performed a structure analysis using ADMIXTURE (*K*, 2–10) (Fig. 3c). At a *K* value of 2 or 3, Group III was one separate cluster, demonstrating that Group III has had a linear evolutionary route due to geographical isolation. Additionally, the genes of Group III gradually penetrated into Group I and Group II accessions. At a *K* value of 3, the accessions were clearly divided into three separate clusters: Group I, Group II, and Group III, which is consistent with the above PCA and phylogenetic tree. Group I and Group II showed a relatively high level of admixture, which may be attributable to a shared ancestor or recent introgression events. To further understand the evolution of the *S. mukorossi* population from the perspective of genomics, we analyzed the decay rate of linkage disequilibrium (LD) of the three subpopulations (indicated by *r*^2^). Based on the one-half *r*^2^ maximum of pairwise SNPs among these three groups, the LD decay rates in Group I, Group II, and Group III were 459 bp (*r*^2^ = .075), 643 bp (*r*^2^ = .0703), and 403 bp (*r*^2^ = .1446), respectively (Fig. 3d). The rapid decline in LD in each population may be due to the high density of SNPs and the high degree of recombination in the *S. mukorossi* genome. Similarly, a study of the quail population structure also revealed a genomic background of low-level LD bird populations, which promote adaptive variation. Genetic diversity (*π*) increased from 5.42 × 10^−3^ in Group I to 5.59 × 10^−3^ in Group II and 6.71 × 10^−3^ in Group III. We also identified the direction of gene flow from Group III (YN) to Group II (HB) to Group I (AH, FJ, JX, GD), indicating that the species in the southwest may be the donor population for *S. mukorossi* planting areas in China (Supplementary Fig. S6). By calculating the fixation index value (*F*_ST_) between these three groups, we found that the *F*_ST_ values were ~0.42 between Group I and Group III populations and ~0.41 between Group II and Group III populations, and the *F*_ST_ between the Group I and Group II populations was only 0.027. These results further indicated that Group I and Group II populations have relatively low genetic differentiation. Moreover, Group I (*π* = 5.42 × 10^−3^) and Group II (*π* = 5.59 × 10^−3^) harbored nearly the same level of genetic diversity with little genetic differentiation (*F*_ST_ = 0.027). Negative mean Tajima’s *D* values between Group I (−0.24) and Group II (−0.41) showed that the *S. mukorossi* population has recently accumulated more low-frequency gene mutations, and regional amplification may have occurred in the population history (Supplementary Fig. 7). Overall, Group I and Group II populations, which existed in close geographical areas, formed one robust clade or a single cluster in structure analyses, with *K* values ranging from 2 to 3, confirming their close genetic relationship.

### Demographic history

To explore the demographic history of *S. mukorossi*, we estimated *N*_e_ using the pairwise sequentially Markovian coalescent (PSMC) method and assumed a mutation rate of 1.25 × 10^−8^ per generation for a generation time of 5 years. The *N*_e_ values of the three groups reflected one dramatic decline period and a recovery period (Fig. 3e). Group I, Group II, and Group III reached the maximum *N*_e_ at ~10 Mya (million years ago) and then began to decline dramatically. One sharp reduction may have resulted from climatic fluctuations corresponding to the start of the Quaternary Ice Age (~2 Mya) [24]. The recovery period occurred ~180 Kya (thousand years ago) during the interglacial period of Marine Isotope Stage 5 (~130 Kya), which may have resulted from a warming climate [39]. According to the results of demographic trajectories, we found that the *N*_e_ values of Group I and Group II were basically identical and higher than that of Group III. The results may be due to relatively low genetic diversity and geographical distributions during the evolution of *S. mukorossi*.

**Figure 4:**
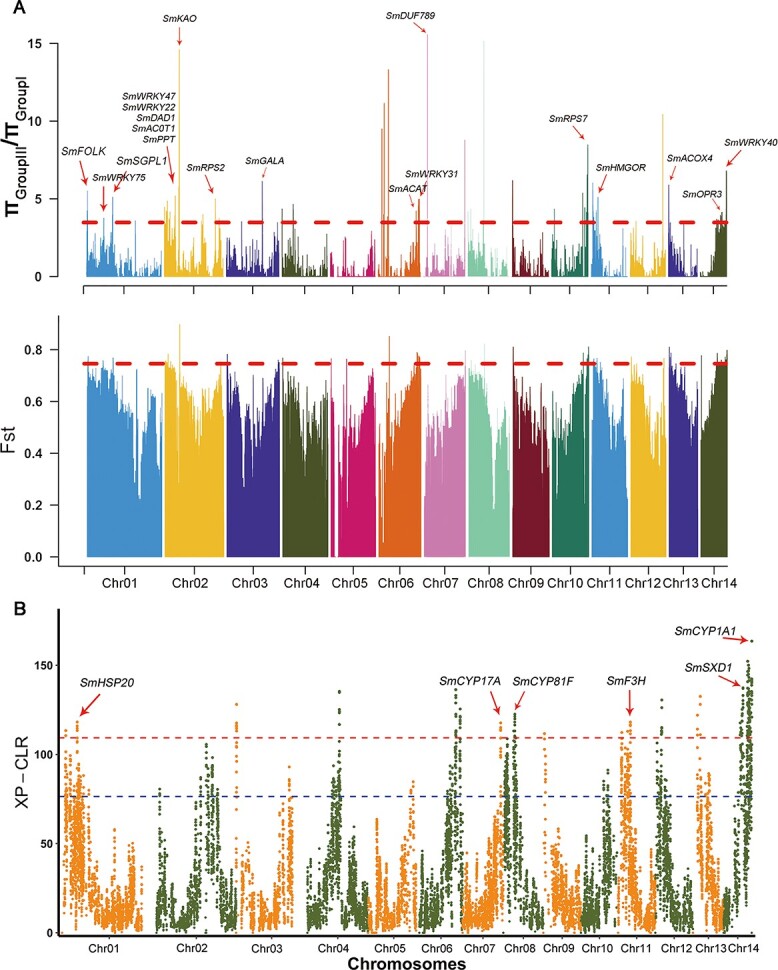
Genome-wide detection and annotation of selective sweeps in 104 *S. mukorossi* accessions. **a** Selective sweeps were identified by π_GroupIII_/π_GroupI_ and *F*_ST_. The red dashed line corresponds to the thresholds for very high significance in the top 5% of the highest values. **b** Manhattan plots of the CLRs among the Group I and Group III *S. mukorossi* populations. Red and blue dashed lines indicate the threshold for the top 1% and top 5% of CLR values, respectively. Genes located within the significant CLR peaks and corresponding annotations are denoted.

**Figure 5:**
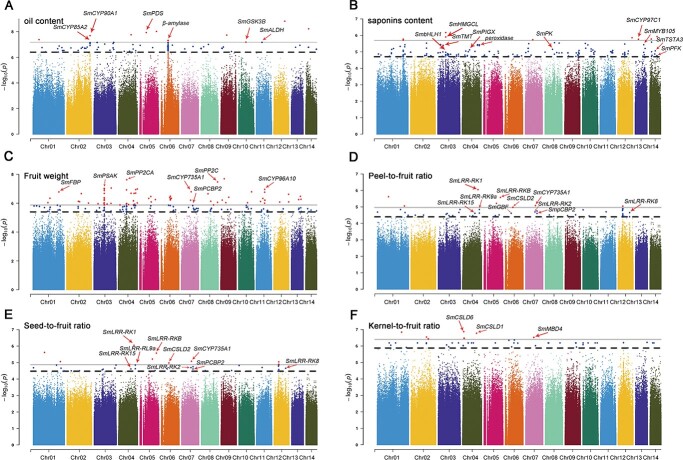
Manhattan plots from GWAS for important agronomic traits in 57 *S. mukorossi* accessions. **a** Oil content. **b** Saponin content. **c** Fruit weight. **d** Peel-to-fruit ratio. **e** Seed-to-fruit ratio. **f** Kernel-to-fruit ratio. Gene names are highlighted in black in candidate regions potentially related to six traits in *S. mukorossi*. Black and gray lines indicate the threshold for the top 5% and top 1% of the highest values, respectively.

### Genome-wide selective sweep signals

It is generally believed that the difference between these three populations lies in their phenotypic characteristics and habitat mainly. In view of the above results, we found that Group I and Group II are closely related and have had gene flow events. To determine the potential divergent selection of these differences, we used three approaches [including the *π* ratio (*π*_GroupIII_/*π*_GroupI_), *F*_ST_, and the cross-population composite likelihood ratio (XP-CLR)] to scan selected regions between the Group I and Group III populations with the top 5% of values. In total, we identified 212 and 149 selective sweeps based on the *π* ratio (>1.25) and *F*_ST_ (>0.42) results, and these selective sweeps harbored 3857 and 4166 genes, respectively (Fig. 4a). GO and KEGG enrichment results showed that these sweep regions were mostly enriched in secondary metabolite biosynthesis, oxidative phosphorylation, plant hormone signal transduction, α-linolenic acid metabolism, and response stimulus (Supplementary Figs S8 and S9; Supplementary Tables S17–S20). To further narrow our list of candidate genes, we found that 2585 candidate genes were shared according to the *π* ratio and *F*_ST_ results. Group I accessions were characterized by the selection of a large number of *RPS* (ribosomal protein S) genes compared with the Group III accessions, including *SmRPS2* (whz_007360), *SmRPS7* (whz_023082), *SmRPS10* (whz_015443), *SmRPS12* (whz_011857), and *SmRPS23* (whz_018422)*. Arabidopsis* homologs containing *RPS3* and *RPS5* have been shown to participate in DNA damage and resistance to *Pseudomonas syringae* [40, 41]. We also used the KEGG database to functionally annotate these 2585 genes, which revealed that the most highly enriched categories were involved in lipid metabolism, signal transduction, environmental adaptation, and terpenoid and polyketide metabolism (Supplementary Fig. S10; Supplementary Table S21). In lipid metabolism, 12 genes were involved in α-linolenic acid metabolism and fatty acid biosynthesis, including *SmDAD1* (whz_020348), *SmACOX4* (whz_006370), *SmOPR3* (whz_020852), *SmACOT1* (whz_026593), *SmFAB2* (whz_000745), *SmgalA* (whz_002077), *SmPPT* (whz_020366), *SmSGPL1* (whz_009676), and *SmACAT* (whz_014940) (Fig. 4a). For terpenoid metabolism, 10 genes were associated with monoterpenoid, diterpenoid, and triterpenoid biosynthesis, including *SmSQLE* (whz_022458), *SmKAO* (whz_020277), *SmGA3ox* (whz_020438), *SmGA2ox* (whz_020996), *SmFOLK* (whz_001194), *SmHMGCR* (whz_016467), and *SmGGPS* (whz_003902) (Fig. 4a). Additionally, 160 TFs were also identified in these sweep regions (*P* < .05), including 30 MYBs, 6 NACs, 13 WRKYs, 5 bZIPs, 11 bHLHs, and 1 AP2 (Supplementary Table S21). NACs and WRKYs have been reported to be involved in defense responses, growth, and development, including *SmNAC2* (whz_006513), *SmNAC75* (whz_020862), *SmNAC83* (whz_006262), *SmWRKY40* (whz_021259), *SmWRKY22* (whz_005902), *SmWRKY47* (whz_006030), *SmWRKY31* (whz_014884), and *SmWRKY75* (whz_009675) [42–45].

Using the data generated from XP-CLR with the top 5% of CLR scores, we identified 163 selective sweep regions that harbored 126 genes and 929 selective sweep regions that harbored 2487 genes that were significantly selected in Group I and Group III (Fig. 4b; Supplementary Fig. S11). Using the data generated from functional annotation analysis, we found that oxidative phosphorylation signaling pathways, including *SmND2* (whz_009672), *SmCOX1* (whz_015024), *SmATP6* (whz_015028), *SmND5* (whz_015033), and *SmPPTC7* (whz_026327), were significantly enriched in Group I but not in Group III. Group III accessions were characterized by the selection of a large number of genes involved in flavonoid and triterpene synthesis, including *SmF3H* (whz_016322), *SmSXD1* (whz_020513), *SmCYP17A* (whz_019898), *SmCYP81F* (whz_018510), *SmCYP1A1* (whz_020835), *SmKAO* (whz_018519), and *SmMYBP* (whz_019890). In addition, we also identified related genes involved in resistance in Group III, including *SmHSP20* (whz_001399), *SmWRKY15* (whz_001652), *SmHSP70* (whz_024119), *SmRPS2* (025316), and *SmRSP19* (whz_019904).

### Genome-wide association studies for six agronomic traits

We used the phenotyping data for six agronomic traits of fruits of 57 *S. mukorossi* samples collected from seven provinces of China, including FJ (17), JX (15), ZJ (6), YN (3), AH (6), HN (5), and GX (5) (Supplementary Table S2). Saponin and oil contents are two important economic traits for superior individuals of *S. mukorossi.* We detected six strong GWAS signals harboring 47 candidate genes, including *SmCYP90A1* (whz_004291), *SmPDS* (whz_013074), *SmGSK3B* (whz_022075), *SmRPS3* (whz_021468), β-amylase (whz_014801), and *SmALDH* (whz_006230) (Fig. 5a; Supplementary Fig. S12). We found that two of the six GWAS signals, located on Chr02 and Chr05, were significantly associated with oil contents, which is consistent with previous reports. For example, two oil-related genes were located in the strong association peaks, including *SmCYP90A1* (whz_004291), which participates in the metabolism of various internal and external substances, such as steroid hormones and fatty acids [46], and *SmGGTS* (whz_017512), which is related to arachidonic acid and cyanoamino acid metabolism [47]. We further detected 73 genes corresponding to six chromosomes (Chr01, Chr03, Chr04, Chr08, Chr13, and Chr14), and a series of genes that have been reported to be significantly associated with the saponin phenotype, including *SmTMT* (whz_003247), *SmCYP97C1* (whz_006615), *SmPIGX* (whz_009575), *SmMYB105* (whz_007070), and *SmbHLH1* transcription factor (whz_003247) (Fig. 5b; Supplementary Fig. S13). In addition, we also identified related genes involved in the metabolism of polysaccharides and flavonoids, including *SmTSTA5* (whz_021561), *SmDFR* (whz_021579) and *SmPK* (whz_019106). *SmAHP* (whz_017177) and *SmPP2C* (whz_017178), which are associated with the fruit weight phenotype, spanning from 5.24 to 5.39 Mb on Chr07 (Fig. 5c; Supplementary Fig. S14). *PP2C* is closely related to abscisic acid signaling, and negatively regulates abscisic acid signaling in the early stages [48]. The *AHP* factor is involved in the signal transduction of cytokinins [49]. GWAS identified a poly(rC)-binding protein 2 gene (*SmPCBP2*, whz_007852) and six leucine-rich repeat receptor kinases (*SmLRR-RK*s), which are associated with the peel-to-fruit ratio and seed-to-fruit ratio (Fig. 5d and e; Supplementary Figs S15 and S16). *SmPCBP2*, as a negative regulator in MAVS-mediated signaling, has been reported to regulate cell growth and death [50]. We performed GWAS for the kernel-to-fruit ratio and found that one SNP located upstream of cellulose synthases (*SmCSLD1*, whz_008313) was strongly associated with the kernel-to-fruit ratio (Fig. 5f; Supplementary Fig. S17). These results provide valuable information for molecular breeding considering genes responsible for fruit weight, the peel-to-fruit ratio, saponin contents, the seed-to-fruit ratio, the kernel-to-fruit ratio, and oil contents in *S. mukorossi*.

## Discussion

To obtain a high-confidence SNP set from resequencing data, a reference genome resource is very important. Several genome assemblies in Sapindaceae have been reported, including *A. yangbiense* (~665 Mb) and *D. longan* Lour. (~471 Mb) [30, 31]. However, no report has involved the genome assembly and annotation of *S. mukorossi*. Diverse chromosome numbers (the chromosome numbers are 11, 13, 14, 15, etc.) and high heterozygosity (1.25%) in Sapindaceae have hindered genome assembly. *S. mukorossi* is an autodiploid with 2*n* = 2*x* = 28 chromosomes based on the results obtained for the molecular karyotype and *K*-mer analysis, differing significantly from the chromosome numbers of previously reported Sapindaceae species (*A. yangbiense*, 2*n* = 2*x* = 26; *D. longan*, 2*n* = 2*x* = 30) [30, 31]. Using the latest sequencing and assembly technology, we obtained the genome of *S. mukorossi* at the chromosome level, revealing a genome size of ~391 Mb with a contig N50 length of 24.66 Mb. By thoroughly mapping resequencing data, 104 accessions generated ~545.83 Gb of clean data and identified 4 351 219 high-quality SNPs and 7 960 863 InDel datasets, yielding the richest genomic resource for further molecular breeding and biological studies of *S. mukorossi* to date.

The resequencing data of the 104 accessions collected from nine provinces of China covered nearly all *S. mukorossi* planting areas. The nucleotide diversity of *S. mukorossi* (7.53 × 10^−3^) was significantly higher than that of other woody plants, such as peach (1.10 × 10^−3^), tea (6.12 × 10^−3^), apple (2.20 × 10^−3^), jujubes (1.73 × 10^−3^), pear (5.50 × 10^−3^), and *Prunus mume* (2.42 × 10^−3^), indicating that the evolutionary process of *S. mukorossi* is characterized by relatively high genetic diversity, which is conducive to adaption to various adverse environments. We further calculated the *F*_ST_ between these three groups and found that the FST of the Group I and Group II accessions, at 0.002, was lower than that of the Group III accessions. These results also suggested that the close relation between accessions from the northeast and south may be attributable to a shared ancestor or recent introgression events due to close geographical areas.

KEGG and GO analyses showed that some of the specific or expanded genes in *S. mukorossi* were significantly enriched in disease resistance, hormone signaling pathways, and lipid metabolism, including *SmRPM1*, *SmRPS2*, acetyl-CoA acyltransferase 1, lipoxygenase and linoleate 9S-lipoxygenase, *Sm*ARR-B, and *Sm*B3. Linoleate 9S-lipoxygenase and lipoxygenase, as key enzymes in linoleic acid and α-linolenic acid metabolism, could enhance key oxylipin synthesis pathways in tillers when upregulated [51]. Expansion of these genes is speculated to affect the accumulation of lipid metabolites. Kernel oil content plays an important role in *S. mukorossi* breeding. Moreover, Group I accessions were also characterized by the selection of a large number of *RPS*s compared with Group III accessions, including *SmRPS2*, *SmRPS4*, *SmRPS7*, *SmRPS10*, *SmRPS12*, and *SmRPS23.* The resistance genes *RPS3*/*5* in *Arabidopsis* mediate resistance to *P. syringae* and DNA damage, and *P. syringae* has been reported to infect many economically important crops [52]. It is speculated that the strong selectivity of these genes enhances environmental adaptability, resulting in better cold tolerance in the northeast than in the southwest. Genome-wide selective sweep analysis revealed that most candidate genes were highly involved in lipid metabolism and terpenoid and polyketide metabolism, including *SmDAD1*, *SmACOX4*, *SmOPR3*, *SmACOT1*, *SmFAB2*, *SmgalA*, *SmPPT*, *SmSGPL1*, *SmACAT*, *SmSQLE*, *SmKAO*, *SmGA3ox*, *SmGA2ox*, *SmFOLK*, *SmHMGCR*, *SmGGPS*, *SmF3H*, *SmCYP17A*, *SmCYP81F*, and *SmCYP1A1*. *SmGA3ox* and *SmGA2ox* are diterpenoid plant hormones with a key role in regulating various processes throughout the life cycles of plants [53]. *SmGGPS* encodes an intermediate in terpenoid biosynthesis that is synthesized by geranylgeranyl diphosphate synthetases [54]. Many studies have focused on the identification of CYP450s for terpenoid biosynthesis, such as CYP716A47, which is related to ginsenoside biosynthesis [55]; CYP76AH1, which catalyzes the turnover of miltiradiene in tanshinone biosynthesis [56]; and CYP71AV14 and CYP71AX30, which are related to sesquiterpene lactone biosynthesis [57]. We speculate that differential selection in these regions may result in richer saponin and oil contents in northeastern accessions than in southwestern accessions, which is consistent with the phenotypic data.

Fruit weight, the peel-to-fruit ratio, saponin contents, the seed-to-fruit ratio, the kernel-to-fruit ratio, and oil contents are important agronomic traits for selecting excellent germplasm resources. To identify potential candidate genes associated with these traits, we performed GWAS using resequencing data from 57 accessions. We found that four peel-to-fruit ratio- and seed-to-fruit ratio-related *LRR-RK* genes have been reported to regulate cell proliferation, stem cell maintenance, hormone perception and other developmental processes in *Arabidopsis* [58]. Similarly, we detected one SNP located upstream of cellulose synthases (*SmCSLD1*, whz_008313) that is responsible for the kernel-to-fruit ratio. *SmCSLD1* is a component of the cell wall biosynthetic machinery and participates in the production of energy [59]. Saponins and oils are also important active compounds in *S. mukorossi*, and different saponin and oil contents may contribute to variation in economic value with respect to breeding. Interestingly, we found that two saponin-related genes were located in strong association peaks, including *SmTMT* (whz_003247), which is involved in ubiquinone and other terpenoid-quinone biosynthesis [60], and *SmbHLH1* transcription factor (whz_003247), which regulates triterpene saponin biosynthesis in *Medicago truncatula* [61]. Our analysis identified candidate genes associated with major agronomic traits, and this information can be used for further reference in the selection of superior germplasm resources. Chromosome-level reference genome assembly and population resequencing data have also substantially expanded basic and applied research for woody trees of Sapindaceae.

## Materials and methods

### Molecular karyotype analysis and genome size estimation of *S. mukorossi*

*S. mukorossi* samples were obtained from Jianou, Nanping, Fujian, China (118.09′20″ E, 27.03′01″ N). Fluorescence *in situ* hybridization and karyotype analysis were carried out according to a previously reported method [62]. The size of the *S. mukorossi* genome was estimated by an optimized DNA flow cytometry method according to Huang *et al*. [63]. The genome size (1C) of *S. mukorossi* (~380 Mb) was estimated to be ~0.43-fold that of tomato (~880 Mb) as an internal reference. Jellyfish software was used to estimate genome size and heterozygosity by 21-*K*-mers with ~21.0 Gb (47×) of clean short reads on the Illumina HiSeq X Ten platform [64].

### Sequencing and assembly of the *S. mukorossi* genome

Genomic DNA was isolated from young leaves using the CTAB method according to a previously reported protocol [65]. One SMRTbell library was constructed and sequenced on a PacBio Sequel II for HiFi CCS. A total of 409.15 Gb of CCS subreads were generated with a read N50 length of 13.45 kb and an average length of 13.16 kb. After trimming and quality control of all reads, ~22.96 Gb of HiFi CCS data were used for genome assembly with hifiasm software (https://github.com/chhylp123/hifiasm), followed by removal of heterozygous sequences with Khaper (https://github.com/lardo/khaper) based on the 21-*K*-mers result. Fresh leaves of *S. mukorossi* were harvested and placed in formaldehyde for 20 min for cross-linking, followed by endonuclease digestion (*Dpn* II), end repair, cyclization, and purification. The captured DNA fragments were used to construct a Hi-C sequencing library. The scaffolds/contigs of the assembled genome sequence were divided, sorted, and oriented into chromosomes using LACHESIS with parameters: CLUSTER_NONINFORMATIVE_RATIO = 1.4, CLUSTER_MAX_LINK_DENSITY = 2.5, CLUSTER_MIN_RE_SITES = 100, ORDER_MIN_N_RES_IN_TRUNK = 60, and ORDER_MIN_N_RES_IN_SHREDS = 60 [66]. Placement and orientation errors exhibiting obvious discrete chromatin interaction patterns were manually adjusted.

### Genome quality assessment

Genome completeness was assessed using the BUSCO databases with the Embryophyta model. High-quality ESTs were mapped to the *S. mukorossi* genome using BLAST (version 0.35) [67]. After trimming and quality control, second-generation data from the Illumina platform were mapped to the genome assembly using Bowtie2 to assess coverage [68].

### Genome annotation

Repeat sequences were identified by combining *de novo* annotation and homology-based methods. For *de novo* annotation, RepeatModeler (version 1.0.4) was used to search for repetitive sequences in the *S. mukorossi* genome, and then the results were used to build a repetitive sequence library [69]. We further used RepeatMasker (version 2.1) to identify repetitive sequences in a previously built repetitive sequence library [70]. For homology-based prediction, a homology-based detection procedure was performed using a conserved BLASTN search in Repbase of RepeatMasker [71]. The repetitive sequence results by *de novo* annotation and homology-based annotation were combined by removing redundant transposable elements. LTR_FINDER (version 1.05) was used to search for intact LTR retrotransposons in the *S. mukorossi* genome [72]. rRNAs and tRNAs were predicted using RNAmmer (version 1.2) and tRNAsan-SE (version 1.23), respectively [73]. In addition, miRNAs and snRNAs were identified using INFERNAL (version 1.1.1) to search the Pfam database [74].

Protein-coding genes were annotated by the MAKER pipeline (version 2.31.9) based on an integrated strategy, including homology-based prediction, RNA-seq data and *ab initio* annotation [75]. For *de novo* gene prediction, we first used BRAKER software to automatically generate full gene structure models with genomic and RNA-seq data [76]. Augustus (version 3.0.3) was applied with the default parameters, and the protein sequences of the training sets used were from the related species *A. yangbiense* and *D. longan* [77]. All of the predicted genes were functionally annotated according to homologous alignments with BLASTP (e-value ≤1e−5, max_target_seqs 1) against the NR, Swiss-Prot, GO, KOG, and KEGG databases [78–82]. TFs were identified by performing BLASTP (e-value ≤1e−5) searches against the plant transcription factor database (PlantTFDB version 5.0).

### Construction of a phylogenetic tree and estimation of gain and loss of gene families

To identify the expanded and contracted *S. mukorossi* gene families, a phylogenetic tree was constructed using orthologous genes for *Punica granatum*, *R. argentea*, *S. oleosum*, *Corymbia citriodora*, *E. grandis*, *R. rubrinervis*, *C. sativa*, *R. chinensis*, *F. vesca*, *M. baccata*, *C. clementina*, *C. unshiu*, *C. sinensis*, *D. longan*, *S. mukorossi*, *A. yangbiense*, *Manihot esculenta*, *Salix dunnii*, *S. brachista*, *P. alba*, and *P. euphratica*. A total of 718 single-copy gene families were shared by the 21 analyzed genomes and used to construct a phylogenetic tree using FastTree (version 2.1.9) and RAxML (version 8.2.12) based on the maximum likelihood method [83]. MCMCtree in PAML (version 4.9) and the TimeTree website (http://www.timetree.org/) were used to estimate the time of species divergence with estimated confidence intervals at the 95% level [84]. CAFE software (version 1.6) was used to calculate the expansion and contraction of gene families [85]. Significantly expanded or contracted genes (*P* ≤ .01) were annotated and enriched by GO and KEGG analyses, respectively.

### Synteny and whole-genome doubling analysis

We analyzed WGD events and determined the source of the homologous genes in *S. mukorossi*. The protein sequences from *S. mukorossi* and other species were searched to identify the best matching pairs using BLASTP (e-value ≤1e−5). Syntenic block analysis was performed using MCScanX with parameters –s 5 –m 5, and *K*s values (synonymous substitution rate) for syntenic gene pairs were calculated using KaKs_Calculator software [86, 87].

### Positively selected genes in *S. mukorossi*

Orthologous gene clusters in *S. mukorossi* and closely related plants were identified using OrthoFinder (version 2.3.12) [88]. The single-copy genes of *S. mukorossi* and closely related species were aligned using MUSCLE [89]. Positive selection sites were detected using Codeml software of the PAML package. The *K*a/*K*s ratios of the single-copy genes were evaluated with a threshold of *K*a/*K*s ≥ 1 and *P* value ≤.05.

### DNA preparation for population sequencing, variant calling and annotation

A total of 104 *S. mukorossi* strains collected from various parts of the world were sequenced with the Illumina HiSeq X Ten platform with an insert size of ~400 bp. DNA of each sample was isolated using the CTAB method according to a previously reported protocol [65]. The complete raw data of the 104 samples were processed for quality control using Trimmomatic with the default parameters [90], and the clean reads were then aligned to *S. mukorossi* using Bwa [91]. The alignments were sorted using SAMtools (version 1.9) [92], and PCR-generated duplicates were removed using Picard (version 2.17). Variant detection was performed for each sample using the GATK (version 3.8.0) HaplotypeCaller tool and subsequently joint-called across all samples using the GATK GenotypeGVCFs tool [92]. To obtain high-quality SNPs, a GATK hard filter was used to filter and merge VCF files with the parameters QD < 2.0 || FS ≤ 60 || MQRankSum< −12.5 || ReadPosRankSum< −8.0 || SOR > 3.0 || MQ < 40.0. The marker set was annotated using SnpEff (version 4.3) with our gene annotation for *S. mukorossi* [93].

### Population genetics analysis

A subset of SNPs from 104 resequencing samples were filtered using PLINK (version 1.9) with the default parameters —geno 0.1, —maf 0.01 [94]. The final SNP set was used to construct a phylogenetic tree using PHYLIP (version 3.2) software based on the neighbor-joining method and implemented within FigTree (version 1.4.2) (http://tree.bio.ed.ac.uk/software/figtree/). PCA of the final SNP set was performed with PLINK (version 1.9), and patterns of genetic variation were visualized using the R package (version 3.4) [95]. To further illustrate the evolutionary history of the *S. mukorossi* genome, a model-based clustering algorithm implemented in ADMIXTURE was used to estimate the relative genome composition for each sample. Population structure analysis was carried out using ADMIXTURE (version 1.3.0), which was run for *K* values from 1 to 10 with 20 bootstrapping replicates [96]. To perform TreeMix analysis, the per-site allele count of each group was calculated using PLINK with the —within command, and the results were then converted into TreeMix input format using the plink2treemix.py script. We then ran TreeMix to construct a maximum-likelihood population tree [97]. LD between pairs of SNPs at up to 100-kb intervals was calculated using PLINK (version 1.9), and PopLDdecay (version 3.26) was used to plot the LD decay graphs by setting the parameter MaxDist to 1000 [98]. To identify candidate regions potentially affected by selection, nucleotide diversity (*π*), and genetic differentiation (*F*_ST_) were calculated using VCFtools (version 0.1.13) in a non-overlapping 100-kb sliding window with a step size of 10 kb [99]. Tajima’s *D* was calculated using VariScan (version 2.0.3) in a non-overlapping 100-kb window with a step size of 10 kb [100].

### Genome-wide selective sweep analysis

Whole-genome detection of selective sweeps was conducted using *F*_ST_ between Group I (47) and Group III (18) populations. To detect the regions under selective sweeps, we used the XP-CLR score to confirm the selective sweeps, with the highest being 5% via the XP-CLR method. The CLR test was implemented in SweepFinder2 for multiple markers across a contiguous grid space [101]. GO term enrichment analysis was performed for candidate genes located within these sweep regions.

### Genome-wide association analysis

We used 57 *S. mukorossi* accessions to perform GWAS on fruit weight, the peel-to-fruit ratio, saponin contents, the seed-to-fruit ratio, the kernel-to-fruit ratio, and oil contents. A subset of SNPs from 57 resequencing samples were filtered using PLINK (version 1.9) with the default parameters —geno 0.1, —maf 0.05 [94]. The final SNP set was used for GWAS by EMMAX and a mixed linear model, and the calculation formula was as follows:

Y = μ + Xb + Zγ + Mu + Pλ + e.

where Y is the observed value vector of the population quantitative trait phenotype; μ is the overall mean; b is the fixed effect vector; γ is the SNP effect vector; u is the polygenic effect vector; λ is the primary weight vector; and X and Z are the correlation matrices corresponding to the fixed effect vectors b and γ, respectively. M is the correlation matrix corresponding to the random effect vector u; P is the correlation matrix corresponding to the primary weight vector λ; and e is the random residual error.

To minimize false-positives, a kinship matrix was estimated with the EMMAX-kin-intel64 program. The Bonferroni threshold for genome-wide significant truncation was set at 0.05/total SNPs. *P* values were used to screen out potential candidate SNPs. Manhattan diagrams and QQ plots were used to represent and evaluate the effect of association analysis, respectively.

## Supplementary Material

Web_Material_uhac012

## Data Availability

Whole-genome sequencing data and resequencing data have been deposited at the National Genomics Data Center, Beijing Institute of Genomics, Chinese Academy of Sciences, under BioProject accession numbers CRA004811, CRA004795 and CRA004820, respectively (https://ngdc.cncb.ac.cn). Whole-genome assembled data have been deposited at the Genome Warehouse of the National Genomics Data Center under accession number GWHBECP00000000 (https://ngdc.cncb.ac.cn/gwh/).
