# Supplementary material for: Chromosome-scale assembly and population diversity analyses provide insights into the evolution of Sapindus mukorossi
Source: Hortic Res. 2022 Feb 17;9:uhac012. doi: 10.1093/hr/uhac012 (PMC8854635; doi:10.1093/hr/uhac012)
Supplement: Web_Material_uhac012 [file web_material_uhac012.zip › Supplementary Tables.pdf]

Supplementary Tables

Table S1 Summary of sequenced *S. mukorossi*.

Sample Acc.	Location	Subpopulation	Longitude	Latitude	Altitude /m	Age /year	Height /m	Diameter /cm	Crown width/m	Canopy transmittance
71	Anhui province, China	Group I	117°16′	31°52′	9	30	9	34	10*10	3
72	Anhui province, China	Group I	117°16′	31°52′	9	15	6	18	6*5	3
73	Anhui province, China	Group I	117°17′	31°52′	7	50	13	39	7*6	3
74	Anhui province, China	Group I	117°12′	31°52′	25	20	6	24	8*7	4
75	Anhui province, China	Group I	116°30′	31°44′	50	12	5	14	3*3	3
69	Anhui province, China	Group I	118°24′	31°29′	24	50	12	45	12*8	4
70	Anhui province, China	Group I	118°22′	31°19′	25	20	6	19	6*5	3
28	Anhui province, China	Group I	118°38′	31°14′	9	20	7	18	6*6	4
29	Anhui province, China	Group I	118°48′	31°16′	10	35	8	31	6*6	4
11	Fujian province, China	Group II	118°08′	27°02′	229	50	19.5	34	17.6	3
63	Fujian province, China	Group II	118°09′	26°59′	194	100	14	61	10*8	3
8	Fujian province, China	Group II	118°07′	26°33′	79	30	12	46	11*12	2
9	Fujian province, China	Group I	118°06′	26°33′	79	30	12	31	8*9	3
10	Fujian province, China	Group I	118°05′	26°34′	80	50	7.5	37	5*6	2
12	Fujian province, China	Group I	118°06′	26°33′	79	20	10	26	8*7	3
66	Fujian province, China	Group I	118°30′	27°55′	248	50	6	31	5*6	3
64	Fujian province, China	Group I	118°22′	27°42′	211	25	11	21/25	10*10	2
65	Fujian province, China	Group I	118°23′	27°44′	251	60	11	52	12*13	3
13	Fujian province, China	Group I	117°32′	26°11′	147	41	10	38	8*7	3
14	Fujian province, China	Group I	117°37′	26°15′	174	30	8	38	5*5	5
4	Fujian province, China	Group I	117°33′	26°11′	88	50	13	41	14*10	4
5	Fujian province, China	Group I	117°33′	26°11′	148	15	5	14	5*4	3
22	Fujian province, China	Group I	116°42′	26°16′	466	35	9	29	7*6	2

25	Fujian province, China	Group I	116°42′	26°16	374	31	7.5	25	3*4	3
27	Fujian province, China	Group I	116°45′	26°15′	368	31	6.2	27	7*7	5
21	Fujian province, China	Group I	116°48′	26°10′	344	41	9	35	9*9	1
23	Fujian province, China	Group I	116°48′	26°10′	344	31	9	40	9*9	3
24	Fujian province, China	Group I	116°48′	26°10′	344	41	13	55	9*9	2
6	Fujian province, China	Group II	117°50′	26°55′	248	31	8	28.7	7*8	3
7	Fujian province, China	Group I	117°52′	26°27′	110	53	15	38.7	8*11	4
1	Fujian province, China	Group I	117°54′	26°47′	110	36	8.4	56	7*9	3
2	Fujian province, China	Group I	117°54′	26°48′	110	36	7	40	6*10	3
3	Fujian province, China	Group I	117°53′	26°57′	130	46	8.2	43	9*8	2
40	Fujian province, China	Group I	117°21′	25° 59′	136	31	15.5	28	12*11	4
100	Guangdong province, China	Group II	114°12′	25°03′	150	100	8	42	8*8	2
101	Guangdong province, China	Group II	114°12′	25°02′	120	100	5	33	5*2	4
106	Guangxi province, China	Group II	110°17′	25°16′	156	100	12	40	10*11	3
107	Guangxi province, China	Group II	110°18′	25°16′	200	80	8	31	11*8	4
108	Guangxi province, China	Group II	109°24′	24°16′	89	100	8	39	6*7	3
109	Guangxi province, China	Group II	109°24′	24°16′	105	100	11	31/37	10*9	2
110	Guangxi province, China	Group II	108°37′	21°56′	-17	40	7	21	6*7	3
111	Guangxi province, China	Group II	109°24′	22°08′	59	100	10	37	7*8	3
112	Guangxi province, China	Group II	109°35′	22°23′	198	50	9	23	8*9	3
103	Guangxi province, China	Group II	111°20′	23°28′	43	200	13	52	15*16	3
104	Guangxi province, China	Group II	111°08′	23°33′	35	60	13	31	8*8	2
76	Hubei province, China	Group II	114°56′	30°12′	433	100	16	28	5*11	2
77	Hubei province, China	Group II	114°56′	30°12′	426	100	12	45	9*7	3
78	Hubei province, China	Group II	114°56′	30°12′	383	100	14	44	8*10	3
81	Hunan province, China	Group II	112°57′	28°10′	39	60	8	34	10*8	3

82	Hunan province, China	Group II	112°57′	28°10′	39	100	10	68	6*9	4
83	Hunan province, China	Group II	112°59′	28°12′	44	100	18	34	10*12	3
84	Hunan province, China	Group II	112°59′	28°12′	44	100	18	37	10*9	3
85	Hunan province, China	Group II	113°29′	27°39′	63	60	8	23	6*12	3
86	Hunan province, China	Group II	113°29′	27°39′	65	50	8	22	7*9	3
97	Jiangxi province, China	Group II	114°39′	25°32′	138	100	12	37	6*9	3
99	Jiangxi province, China	Group II	114°32′	25°27′	157	260	10	47	8*6	3
98	Jiangxi province, China	Group II	114°34′	25°32′	150	300	11	21/25	11*10	3
96	Jiangxi province, China	Group II	114°45′	25°39′	136	100	12	57	8*7	3
36	Jiangxi province, China	Group II	116°10′	28°20′	50	52	12	56	10*11	3
39	Jiangxi province, China	Group II	116°08′	28° 21′	50	45	13.5	36	10*12	2
37	Jiangxi province, China	Group I	115°46′	29°12′	55	6	7	12	10*12	2
38	Jiangxi province, China	Group I	115°48′	29°26′	837	21	9	23	4*4	4
90	Jiangxi province, China	Group I	115°54′	28°41′	62	70	11	23	4*5	3
91	Jiangxi province, China	Group I	116°00′	25°58′	236	450	20	64	12*14	3
92	Jiangxi province, China	Group II	116°00′	25°57′	236	300	13	55	3*4	4
15	Jiangxi province, China	Group II	114°34′	26°22′	106	80	12	41	11*13	3
16	Jiangxi province, China	Group II	114°03′	26°20′	130	53	14	55	9*8	3
93	Jiangxi province, China	Group II	114°53′	26°40′	126	51	12.5	32/38	10*9	2
94	Jiangxi province, China	Group II	114°34′	26°22′	105	100	13	33	10*8	2
95	Jiangxi province, China	Group II	114°31′	26°20′	125	10	6	16	6*7	3
88	Jiangxi province, China	Group I	114°55	27°48′	69	40	5	21	4*4	3
89	Jiangxi province, China	Group II	114°54′	27°49′	65	50	8	30	6*8	3
87	Jiangxi province, China	Group II	114°23′	27°49′	98	50	6	23	3*3	2
26	Jiangxi province, China	Group II	117°01′	28°14′	148	30	8	16	4*3	3
44	Yunnan province, China	Group III	102°53′	23°44′	1384	>100	15.8	78	12*10	3

46	Yunnan province, China	Group III	102°53′	23°44′	1384	>100	12	48	11*10	3
113	Yunnan province, China	Group III	102°54′	23°43′	1384	40	7	32	3*5	2
116	Yunnan province, China	Group III	102°54′	23°43′	1384	30	15	28	5*4	3
41	Yunnan province, China	Group III	102°44′	25°08′	1944	430	15	72	7*6	2
42	Yunnan province, China	Group III	102°44′	25°08′	1944	>400	18	131	10*8	3
43	Yunnan province, China	Group III	102°44′	25°08′	1944	>400	18	73	6*8	3
56	Yunnan province, China	Group III	101°02′	23°04′	1320	>80	14	56	9*8	3
57	Yunnan province, China	Group III	101°02′	23°03′	1365	>80	16	48	10*12	3
48	Yunnan province, China	Group III	102°38′	23°38′	1469	>45	14	36	9*7	3
49	Yunnan province, China	Group III	102°38′	23°38′	1469	>45	12.5	36	8*5	2
50	Yunnan province, China	Group III	102°22′	23°27′	1693	>260	14	89	16*15	3
51	Yunnan province, China	Group III	102°22′	23°27′	1671	>400	13	101	16*15	3
54	Yunnan province, China	Group III	102°22′	23°27′	1565	>300	17	69	10*8	2
117	Yunnan province, China	Group III	102°22′	23°27′	1693	50	15	52	8*12	3
59	Yunnan province, China	Group III	101°53′	22°05′	749	>50	13	52	6*8	3
62	Yunnan province, China	Group III	101°15′	21°55′	606	>35	17	31	6*6	3
60	Fujian province, China	Group I	101°55′	21°55′	546	>45	18	45	15*14	3
61	Yunnan province, China	Group III	101°55′	21°55′	546	>50	18	30.28	14*13	3
30	Zhejiang province, China	Group I	120°07′	30°15′	10	36	11	27	8*7	3
31	Zhejiang province, China	Group I	120°07′	30°15′	10	35	10	22	8*7	3
32	Zhejiang province, China	Group I	120°07′	30°15′	10	45	10	35	7*8	5
33	Zhejiang province, China	Group I	120°07′	30°15′	10	36	9	25	6*8	4
34	Zhejiang province, China	Group I	120°07′	30°15′	10	31	11	30	7*9	3
35	Zhejiang province, China	Group I	120°07′	30°15′	10	45	10	43	8*9	4
19	Zhejiang province, China	Group I	119°39′	29°06′	46	35	13.5	36	9*16	4
20	Zhejiang province, China	Group I	119°39′	29°06′	25	30	10	28	7*8	3

68	Zhejiang province, China	Group I	119°35′	29°09′	73	300	5.5	33	4*5	4
17	Zhejiang province, China	Group I	118°52′	28°57′	94	108	17.9	64	10*10	4
18	Zhejiang province, China	Group I	118°52′	28°57′	95	28	9	30	8*9	3

Table S2 Summary of sequenced 57 *S. mukorossi* for GWAS.

Sample Acc.	fruit weight/g	peel-to-fruit ratio	saponins content/%	seed-to-fruit ratio	kernel-to-fruit ratio	oil content/%
1	3.04	0.53	73.14	0.47	0.3104	43.93
2	1.88	0.46	80.82	0.54	0.2993	40.74
3	3.03	0.63	65.79	0.37	0.2472	38.82
5	3.44	0.55	59.98	0.45	0.3183	38.65
6	1.92	0.53	81.64	0.47	0.3261	38.96
8	3.63	0.67	74.95	0.33	0.2561	32.95
13	3.41	0.58	78.18	0.42	0.3323	38.04
14	3.11	0.57	64.17	0.43	0.2931	39.54
15	3.59	0.59	44.05	0.41	0.2418	39.55
16	2.16	0.54	56.59	0.46	0.2313	41.56
17	3.19	0.62	69.07	0.38	0.2567	40.18
18	2.24	0.58	59.94	0.42	0.2166	36.84
19	3.71	0.61	75.83	0.39	0.2458	36.65
20	3.69	0.59	61.58	0.41	0.3197	41.63
22	3.09	0.64	41.53	0.36	0.2541	40.93
24	5.34	0.65	40.75	0.35	0.3002	37.11
25	2.93	0.68	72.48	0.32	0.2252	44.28
26	4.51	0.64	54.95	0.36	0.2627	34.44
27	3.34	0.67	74.98	0.33	0.2458	40.78
33	3.24	0.52	49.05	0.48	0.27	35.49
36	2.93	0.59	59.03	0.41	0.2804	35.06
37	2.29	0.61	43.33	0.39	0.2342	34.71
40	3.11	0.57	59.94	0.43	0.2983	40.72
43	2.24	0.51	77.45	0.49	0.4229	40.56
56	6.35	0.69	31.64	0.31	0.2786	38.70
63	2.71	0.54	71.6	0.46	0.2416	33.69
64	2.79	0.63	54.84	0.37	0.2767	34.81
65	2.42	0.53	73.33	0.47	0.2483	34.07
66	3.28	0.61	49.17	0.39	0.273	38.82
68	3.56	0.62	48.22	0.38	0.3159	34.74
70	4	0.5	81.56	0.5	0.3646	33.30
71	3.16	0.5	56.63	0.5	0.2508	43.06
72	3.91	0.59	72.4	0.41	0.2883	36.83
73	3.52	0.57	62.23	0.43	0.3012	40.25
74	3.88	0.57	63.27	0.43	0.1662	41.73
75	2.03	0.49	71.62	0.51	0.2869	37.46
81	2.1	0.49	54.13	0.51	0.2648	35.51
82	4.28	0.62	64.06	0.38	0.2133	37.39
85	2.68	0.51	69.1	0.49	0.1692	37.40

86	3.04	0.59	65.66	0.41	0.2164	44.37
87	2.32	0.59	63.16	0.41	0.1718	46.08
89	2.12	0.51	67.43	0.49	0.1563	42.86
90	3.28	0.69	59.86	0.31	0.2936	32.20
91	2.62	0.59	57.27	0.41	0.1468	33.46
93	2.37	0.54	53.28	0.46	0.1447	37.70
94	2.73	0.5	58.29	0.5	0.2773	39.26
96	3.67	0.58	61.61	0.42	0.2773	38.20
97	2.95	0.61	61.54	0.39	0.2662	35.04
98	3.44	0.61	59.94	0.39	0.2049	58.6
99	3.11	0.61	57.37	0.39	0.2116	36.87
106	3.01	0.64	53.26	0.36	0.1974	33.43
107	3.25	0.64	47.45	0.36	0.1935	33.4
108	4.39	0.65	80.65	0.35	0.2093	41.22
110	4.15	0.64	44.15	0.36	0.2605	36.16
112	4.17	0.56	34.92	0.44	0.3125	38.85
113	3.43	0.64	35.79	0.36	0.239	33.75
83	3.62	0.61	63.33	0.39	0.2066	33.40

Table S3 Sequencing data used for *S. mukorossi* genome construction. Note that the sequence coverage was calculated using the Kmer -based estimated genome size.

Library resource	Sequencing platform	Insert size (bp)	Clean data (Gb)	Sequence coverage (X)	Use of the data
Genome	Illumina HiSeq X Ten	250 bp	21	47	Genome estimation
Genome	PacBio HiFi	20 kb	22.96	57.4	Genome assembly
Hi-C	Illumina HiSeq X Ten	250 bp	40.96	102	Chromosome construction
mRNA	NovaSeq 6000	150 bp	54	-	Annotation
Requencing data	Illumina HiSeq X Ten	150 bp	545.83	-	Population genetic analysis and GWAS analysis

Table S4 Assembly statistics for the *S. mukorossi* genome

Items	Hifiasm		Hi-C	
	Contig_len(Mb)	Contig_number	Scaffold_len(Mb)	Scaffold_number
Total	391.55	1112	391.57	1007
Max	53.08	-	44.80	-
Number>=2 kb	-	1112	-	1007
N50	2.88	-	24.66	-

Table S5 Pseudomolecule length statistics after Hi-C assisted assembly

GroupID	Length	Scaf Num
LG01	44800840	30
LG02	35436386	19
LG03	31624267	25
LG04	27116659	15
LG05	25299562	9
LG06	24845014	29
LG07	24662827	18
LG08	21895147	8
LG09	21881448	16
LG10	21611680	6
LG11	21130090	12
LG12	19383384	8
LG13	17294447	42
LG14	15756312	5
Total	352738063	242
Orangin Genome	391552161	
Loading Rate	0.900871194528	

Table S6 Assessment of the completeness of the *S. mukorossi* genome assembly by BUSCO

Type	Number	Percent (%)
Complete BUSCOs (C)	356	83.8
Complete and single-copy BUSCOs (S)	351	82.6
Complete and duplicated BUSCOs (D)	5	1.2
Fragmented BUSCOs (F)	7	1.6
Missing BUSCOs (M)	62	14.6
Total BUSCO groups searched	425	100

Table S7 Transcriptome reads alignment rate of the *S. mukorossi* genome

Total_EST_Num:	659372
Total_EST_Len:	557124883
Bases_Covered_By_Assembly:	0.940969657506915
Seq_Covered_By_Assembly:	0.402437470805554
Num90:	230022
Per90:	0.348850117991058
Num50:	250017
Per50:	0.37917442657559

Table S8 Annotation statistics for the *S. mukorossi* genome

Annotation statistics for the genome	Number	Percent (%)
Total protein	31,853	
NR	27,335	85.82
Swiss-Prot	23,766	74.61
GO	18,615	58.44
KOG	14,452	45.37
KEGG	11,025	34.61
In all database	6,685	20.99
In at least one database	27,509	86.36

Table S9 Transcription factors

Number	Class
850	protein kinase family protein
424	pentatricopeptide (PPR) repeat-containing protein
132	Disease resistance protein (TIR-NBS-LRR class)
93	basic helix-loop-helix (bHLH) DNA-binding superfamily protein
53	Integrase-type DNA-binding superfamily protein
42	Homeodomain-like superfamily protein
40	AP2/B3-like transcriptional factor family protein
38	DNAJ heat shock N-terminal domain-containing protein
62	myb-like transcription factor family protein
29	Duplicated homeodomain-like superfamily protein
24	WD-40 repeat family protein / zfwd4 protein (ZFWD4)
24	Zinc finger C-x8-C-x5-C-x3-H type family protein
22	C2H2-like zinc finger protein
21	Basic-leucine zipper (bZIP) transcription factor family protein
21	GRAS family transcription factor
19	C2H2 and C2HC zinc fingers superfamily protein
35	AGAMOUS-like 62
27	NAC-domain protein 101
16	B-box type zinc finger protein with CCT domain
16	K-box region and MADS-box transcription factor family protein
14	zinc finger WD40 repeat protein 1
12	NAC (No Apical Meristem) domain transcriptional regulator superfamily protein
12	myb-like HTH transcriptional regulator family protein
12	nucleic acid binding;zinc ion binding;DNA binding
12	zinc finger (C2H2 type) family protein
11	AGAMOUS-like 103
11	auxin response factor 1
10	Calmodulin-binding transcription activator protein with CG-1 and Ankyrin domains
10	Dof-type zinc finger DNA-binding family protein
8	Far-red impaired responsive (FAR1) family protein
8	MADS-box transcription factor family protein
8	Transcriptional factor B3 family protein / auxin-responsive factor AUX/IAA-related
8	auxin response factor 9
8	bZIP transcription factor family protein
8	sequence-specific DNA binding transcription factors

Table S10 Repetitive element annotations in the *S. mukorossi*.

	number of elements*	length occupied	percentage of sequence
Retroelements	38863	36299360 bp	9.27 %
SINEs:	245	13341 bp	0.00 %
Penelope	181	17011 bp	0.00 %
LINEs:	4163	1402605 bp	0.36 %
CRE/SLACS	13	993 bp	0.00 %
L2/CR1/Rex	526	31990 bp	0.01 %
R1/LOA/Jockey	189	8811 bp	0.00 %
R2/R4/NeSL	56	10121 bp	0.00 %
RTE/Bov-B	60	4235 bp	0.00 %
L1/CIN4	2811	1308016 bp	0.33 %
LTR elements:	34455	34883414 bp	8.91 %
BEL/Pao	101	12966 bp	0.00 %
Ty1/Copia	10514	11218738 bp	2.87 %
Gypsy/DIRS1	22239	23310071 bp	5.95 %
Retroviral	378	18787 bp	0.00 %
DNA transposons	15751	2904112 bp	0.74 %
hobo-Activator	4787	1282053 bp	0.33 %
Tc1-IS630-Pogo	924	129162 bp	0.03 %
En-Spm	0	0 bp	0.00 %
MuDR-IS905	0	0 bp	0.00 %
PiggyBac	30	1643 bp	0.00 %
Tourist/Harbinger	1621	372303 bp	0.10 %
Other (Mirage, P-element, Transib)	283	16802 bp	0.00 %
Rolling-circles	0	0 bp	0.00 %
Unclassified:	1877	518423 bp	0.13 %
Total interspersed repeats:		39721895 bp	10.14 %
Small RNA:	4717	5339089 bp	1.36 %
Satellites:	370	32260 bp	0.01 %
Simple repeats:	233495	9615880 bp	2.46 %
Low complexity:	47729	2513748 bp	0.64 %

Table S11 Statistical analysis of non-coding RNAs in *S. mukorossi.*

Class	number	totalLen	meanLen
Cis-reg;	8	315	39.375
Cis-reg; riboswitch;	1	134	134
Gene;	4	1175	293.75
Gene; antisense;	119	22236	186.8571429
Gene; miRNA;	88	11427	129.8522727
Gene; rRNA;	3035	5755584	1896.403295
Gene; ribozyme;	3	761	253.6666667
Gene; sRNA;	1	389	389
Gene; snRNA; snoRNA; CD-box;	464	48196	103.8706897
Gene; snRNA; snoRNA; HACA-box;	29	3563	122.862069
Gene; snRNA; splicing;	79	11394	144.2278481
Gene; tRNA;	4018	303873	75.62792434
Intron;	2675	281335	105.1719626

Table S12 KEGG enrichment of expanded genes.

KEGG_B_class	Pathway	out
Energy metabolism	Oxidative phosphorylation	577
Energy metabolism	Photosynthesis	541
Transcription	RNA polymerase	177
Global and overview maps	Metabolic pathways	1027
Translation	Ribosome	588
Energy metabolism	Carbon fixation pathways in prokaryotes	41
Carbohydrate metabolism	Propanoate metabolism	41
Lipid metabolism	Fatty acid biosynthesis	41
Cell growth and death	Cell cycle - Caulobacter	54
Nervous system	Retrograde endocannabinoid signaling	44
Neurodegenerative diseases	Parkinson disease	61
Carbohydrate metabolism	Pyruvate metabolism	41
Global and overview maps	Fatty acid metabolism	41
Energy metabolism	Carbon fixation in photosynthetic organisms	52
Carbohydrate metabolism	Glyoxylate and dicarboxylate metabolism	52
Global and overview maps	Carbon metabolism	93
Neurodegenerative diseases	Huntington disease	64
Neurodegenerative diseases	Alzheimer disease	64
Environmental adaptation	Thermogenesis	61
Aging	Longevity regulating pathway - worm	54
Endocrine and metabolic diseases	Non-alcoholic fatty liver disease (NAFLD)	24
Global and overview maps	Microbial metabolism in diverse environments	93
Cellular community - eukaryotes	Gap junction	3
Circulatory system	Cardiac muscle contraction	3
Metabolism of terpenoids and polyketides	Zeatin biosynthesis	2
Transcription	Basal transcription factors	2
Replication and repair	Nucleotide excision repair	2
Amino acid metabolism	Histidine metabolism	1
Signal transduction	Ras signaling pathway	3
Transport and catabolism	Phagosome	3
Endocrine system	Prolactin signaling pathway	1
Endocrine system	Ovarian Steroidogenesis	1
Signal transduction	Two-component system	3
Lipid metabolism	Steroid hormone biosynthesis	1
Endocrine and metabolic diseases	Cushing syndrome	1
Global and overview maps	Biosynthesis of secondary metabolites	96
Global and overview maps	Biosynthesis of amino acids	1
Signal transduction	Plant hormone signal transduction	1
Environmental adaptation	Plant-pathogen interaction	2
Infectious diseases	Pathogenic Escherichia coli infection	3

Table S13 GO enrichment of expanded genes.

GO ID (level1)	GO Term (level1)	GO Term (level2)	number_of_out
GO:0005575	Cellular Component	organelle	30
GO:0005575	Cellular Component	cell	30
GO:0005575	Cellular Component	cell part	30
GO:0005575	Cellular Component	organelle part	18
GO:0005575	Cellular Component	membrane	14
GO:0008150	Biological Process	metabolic process	3
GO:0008150	Biological Process	cellular process	3
GO:0005575	Cellular Component	synapse part	2
GO:0005575	Cellular Component	synapse	2
GO:0003674	Molecular Function	binding	1
GO:0003674	Molecular Function	structural molecule activity	1
GO:0005575	Cellular Component	membrane-enclosed lumen	1
GO:0005575	Cellular Component	macromolecular complex	1

Table S14 KEGG enrichment of contracted genes (Top 30).

KEGG_B_class	Pathway	out
Global and overview maps	Metabolic pathways	83
Global and overview maps	Biosynthesis of secondary metabolites	52
Environmental adaptation	Plant-pathogen interaction	21
Global and overview maps	Microbial metabolism in diverse environments	17
Global and overview maps	Carbon metabolism	16
Translation	Ribosome	12
Transcription	Spliceosome	11
Biosynthesis of other secondary metabolites	Phenylpropanoid biosynthesis	11
Global and overview maps	Biosynthesis of amino acids	11
Nucleotide metabolism	Purine metabolism	8
Carbohydrate metabolism	Starch and sucrose metabolism	8
Signal transduction	Plant hormone signal transduction	8
Infectious diseases	Salmonella infection	7
Carbohydrate metabolism	Glycolysis / Gluconeogenesis	6
Infectious diseases	Pathogenic Escherichia coli infection	6
Infectious disease: viral	Human immunodeficiency virus 1 infection	6
Folding, sorting and degradation	Protein processing in endoplasmic reticulum	6
Carbohydrate metabolism	Glyoxylate and dicarboxylate metabolism	6
Environmental adaptation	Thermogenesis	6
Endocrine system	Glucagon signaling pathway	5
Signal transduction	FoxO signaling pathway	5
Transport and catabolism	Peroxisome	5
Signal transduction	AMPK signaling pathway	5
Cell growth and death	Cellular senescence	5
Immune system	Toll and Imd signaling pathway	5
Signal transduction	MAPK signaling pathway - plant	5
Cell growth and death	Oocyte meiosis	5
Infectious diseases	Herpes simplex infection	5
Folding, sorting and degradation	Ubiquitin mediated proteolysis	5

Table S15 KEGG enrichment of specific gene (Top 30).

KEGG_B_class	Pathway	out
Global and overview maps	Metabolic pathways	1541
Translation	Ribosome	1467
Energy metabolism	Photosynthesis	971
Energy metabolism	Oxidative phosphorylation	687
Global and overview maps	Biosynthesis of secondary metabolites	179
Transcription	RNA polymerase	121
Global and overview maps	Microbial metabolism in diverse environments	119
Global and overview maps	Carbon metabolism	116
Energy metabolism	Carbon fixation in photosynthetic organisms	92
Carbohydrate metabolism	Glyoxylate and dicarboxylate metabolism	88
Aging	Longevity regulating pathway - worm	87
Cell growth and death	Cell cycle - Caulobacter	81
Global and overview maps	Biosynthesis of amino acids	30
Infectious diseases	Salmonella infection	25
Environmental adaptation	Plant-pathogen interaction	24
Translation	RNA transport	21
Infectious diseases	Shigellosis	17
Cell growth and death	Cell cycle	17
Infectious disease: viral	Human immunodeficiency virus 1 infection	17
Global and overview maps	Fatty acid metabolism	16
Neurodegenerative diseases	Huntington disease	16
Lipid metabolism	Fatty acid biosynthesis	15
Folding, sorting and degradation	Protein processing in endoplasmic reticulum	15
Neurodegenerative diseases	Alzheimer disease	15
Infectious diseases	Pathogenic Escherichia coli infection	15
Cancers	Pathways in cancer	15
Carbohydrate metabolism	Pyruvate metabolism	14
Biosynthesis of other secondary metabolites	Phenylpropanoid biosynthesis	14
Cell growth and death	Cell cycle - yeast	14

Table S16 KEGG enrichment of positively selected genes.

ID	KO	Kegg	Pathway	Class
whz_000032	K03883	ko00190	Oxidative phosphorylation	Metabolism
whz_000032	K03883	ko04723	Retrograde endocannabinoid signaling	Organismal Systems
whz_000032	K03883	ko04714	Thermogenesis	Organismal Systems
whz_000032	K03883	ko05012	Parkinson disease	Human Diseases
whz_000208	K14491	ko04075	Plant hormone signal transduction	Environmental Information Processing
whz_000548	K14508	ko04075	Plant hormone signal transduction	Environmental Information Processing
whz_001817	K01183	ko00520	Amino sugar and nucleotide sugar metabolism	Metabolism
whz_003329	K13459	ko04626	Plant-pathogen interaction	Organismal Systems
whz_004052	K03361	ko04120	Ubiquitin mediated proteolysis	Genetic Information Processing
whz_004052	K03361	ko04111	Cell cycle - yeast	Cellular Processes
whz_004052	K10260	ko04120	Ubiquitin mediated proteolysis	Genetic Information Processing
whz_004053	K03361	ko04120	Ubiquitin mediated proteolysis	Genetic Information Processing
whz_004053	K03361	ko04111	Cell cycle - yeast	Cellular Processes
whz_004053	K10260	ko04120	Ubiquitin mediated proteolysis	Genetic Information Processing
whz_004777	K02703	ko00195	Photosynthesis	Metabolism
whz_004777	K03243	ko03013	RNA transport	Genetic Information Processing
whz_006074	K03879	ko00190	Oxidative phosphorylation	Metabolism
whz_006074	K03879	ko04723	Retrograde endocannabinoid signaling	Organismal Systems
whz_006074	K03879	ko04714	Thermogenesis	Organismal Systems
whz_006074	K03879	ko05012	Parkinson disease	Human Diseases
whz_006095	K03879	ko00190	Oxidative phosphorylation	Metabolism
whz_006095	K03879	ko04723	Retrograde endocannabinoid signaling	Organismal Systems
whz_006095	K03879	ko04714	Thermogenesis	Organismal Systems
whz_006095	K03879	ko05012	Parkinson disease	Human Diseases

Table S17 GO term enrichment for sweep regions based on the results of Fst.

GO Term (level1)	GO ID (level2)	GO Term (level2)	number_of_out (All)
Cellular Component	GO:0005623	cell	51
Cellular Component	GO:0044464	cell part	51
Cellular Component	GO:0043226	organelle	50
Cellular Component	GO:0044422	organelle part	32
Cellular Component	GO:0016020	membrane	30
Biological Process	GO:0008152	metabolic process	6
Biological Process	GO:0009987	cellular process	6
Biological Process	GO:0044699	single-organism process	4
Biological Process	GO:0050896	response to stimulus	3
Molecular Function	GO:0003824	catalytic activity	3
Cellular Component	GO:0032991	macromolecular complex	3
Biological Process	GO:0051179	localization	2
Molecular Function	GO:0005198	structural molecule activity	2
Cellular Component	GO:0044425	membrane part	2
Cellular Component	GO:0044456	synapse part	2
Cellular Component	GO:0045202	synapse	2
Biological Process	GO:0032501	multicellular organismal process	1
Biological Process	GO:0032502	developmental process	1
Biological Process	GO:0040011	locomotion	1
Biological Process	GO:0050789	regulation of biological process	1
Biological Process	GO:0065007	biological regulation	1
Biological Process	GO:0071840	cellular component organization or biogenesis	1
Molecular Function	GO:0005215	transporter activity	1
Molecular Function	GO:0005488	binding	1
Cellular Component	GO:0030054	cell junction	1
Cellular Component	GO:0031974	membrane-enclosed lumen	1
Cellular Component	GO:0055044	symplast	1

Table S18 KEGG pathway enrichment for sweep regions based on the results of Fst (Top 30).

KEGG_B_class	Pathway	out	Pvalue	Qvalue	Pathway
Global and overview maps	Metabolic pathways	398	0.9937663	1.00E+00	ko01100
Global and overview maps	Biosynthesis of secondary metabolites	179	3.31E-09	1.65E-07	ko01110
Energy metabolism	Oxidative phosphorylation	83	1	1.00E+00	ko00190
Environmental adaptation	Thermogenesis	73	2.00E-18	5.52E-16	ko04714
Neurodegenerative diseases	Huntington disease	71	1.28E-16	1.49E-14	ko05016
Neurodegenerative diseases	Alzheimer disease	71	3.17E-16	2.76E-14	ko05010
Translation	Ribosome	66	1	1.00E+00	ko03010
Neurodegenerative diseases	Parkinson disease	62	3.17E-18	5.52E-16	ko05012
Global and overview maps	Carbon metabolism	47	0.0775807	1.43E-01	ko01200
Signal transduction	Plant hormone signal transduction	45	7.95E-05	1.20E-03	ko04075
Nervous system	Retrograde endocannabinoid signaling	40	1.71E-12	1.19E-10	ko04723
Global and overview maps	Microbial metabolism in diverse environments	40	0.6804587	7.49E-01	ko01120
Infectious diseases	Salmonella infection	39	6.97E-05	1.15E-03	ko05132
Environmental adaptation	Plant-pathogen interaction	36	0.1525421	2.37E-01	ko04626
Endocrine and metabolic diseases	Non-alcoholic fatty liver disease (NAFLD)	32	1.22E-10	7.10E-09	ko04932
Carbohydrate metabolism	Pentose and glucuronate interconversions	31	7.63E-07	2.95E-05	ko00040
Folding, sorting and degradation	Protein processing in endoplasmic reticulum	31	0.000949682	6.49E-03	ko04141
Global and overview maps	Biosynthesis of amino acids	31	0.006641379	2.74E-02	ko01230
Infectious disease: viral	Human immunodeficiency virus 1 infection	28	0.004444804	2.18E-02	ko05170
Infectious diseases	Pathogenic Escherichia coli infection	27	0.000532038	4.73E-03	ko05130
Infectious diseases	Tuberculosis	26	0.000481729	4.53E-03	ko05152
Transport and catabolism	Endocytosis	25	0.00154457	9.28E-03	ko04144
Translation	RNA transport	25	0.004565632	2.21E-02	ko03013
Carbohydrate metabolism	Starch and sucrose metabolism	24	0.00178827	1.04E-02	ko00500
Folding, sorting and degradation	Ubiquitin mediated proteolysis	23	0.000242308	2.72E-03	ko04120
Infectious diseases	Epstein-Barr virus infection	23	0.003496646	1.84E-02	ko05169
Nervous system	Neurotrophin signaling pathway	22	0.00119081	7.67E-03	ko04722
Signal transduction	Two-component system	21	5.21E-07	2.26E-05	ko02020
Infectious diseases	Shigellosis	21	0.000157304	2.03E-03	ko05131

Table S19 GO term enrichment for sweep regions based on the results of π ratio ($\pi_{GroupIII}/\pi_{GroupI}$).

GO Term (level1)	GO ID (level2)	GO Term (level2)	number_of_out (All)
Cellular Component	GO:0005623	cell	51
Cellular Component	GO:0044464	cell part	51
Cellular Component	GO:0043226	organelle	50
Cellular Component	GO:0044422	organelle part	32
Cellular Component	GO:0016020	membrane	30
Biological Process	GO:0008152	metabolic process	6
Biological Process	GO:0009987	cellular process	6
Biological Process	GO:0044699	single-organism process	4
Biological Process	GO:0050896	response to stimulus	3
Molecular Function	GO:0003824	catalytic activity	3
Cellular Component	GO:0032991	macromolecular complex	3
Biological Process	GO:0051179	localization	2
Molecular Function	GO:0005198	structural molecule activity	2
Cellular Component	GO:0044425	membrane part	2
Cellular Component	GO:0044456	synapse part	2
Cellular Component	GO:0045202	synapse	2
Biological Process	GO:0032501	multicellular organismal process	1
Biological Process	GO:0032502	developmental process	1
Biological Process	GO:0040011	locomotion	1
Biological Process	GO:0050789	regulation of biological process	1
Biological Process	GO:0065007	biological regulation	1
Biological Process	GO:0071840	cellular component organization or biogenesis	1
Molecular Function	GO:0005215	transporter activity	1
Molecular Function	GO:0005488	binding	1
Cellular Component	GO:0030054	cell junction	1
Cellular Component	GO:0031974	membrane-enclosed lumen	1
Cellular Component	GO:0055044	symplast	1

Table S20 KEGG pathway enrichment for sweep regions based on the results of π ratio ($\pi_{GroupIII}/\pi_{GroupI}$) (Top30)

KEGG_B_class	Pathway	out	Pvalue	Qvalue	Pathway
Global and overview maps	Metabolic pathways	164	0.9991305	1.00E+00	ko01100
Global and overview maps	Biosynthesis of secondary metabolites	65	0.05425978	2.55E-01	ko01110
Translation	Ribosome	64	0.9999931	1.00E+00	ko03010
Energy metabolism	Oxidative phosphorylation	52	0.9435328	1.00E+00	ko00190
Neurodegenerative diseases	Alzheimer disease	38	5.36E-11	7.02E-09	ko05010
Environmental adaptation	Thermogenesis	35	1.05E-09	8.50E-08	ko04714
Neurodegenerative diseases	Huntington disease	35	2.00E-09	1.22E-07	ko05016
Neurodegenerative diseases	Parkinson disease	32	5.76E-11	7.02E-09	ko05012
Environmental adaptation	Plant-pathogen interaction	29	9.45E-05	2.10E-03	ko04626
Signal transduction	Plant hormone signal transduction	25	0.000170131	2.77E-03	ko04075
Translation	RNA transport	22	4.75E-07	2.32E-05	ko03013
Infectious diseases	Salmonella infection	21	0.000405034	5.20E-03	ko05132
Infectious disease: viral	Human immunodeficiency virus 1 infection	20	6.11E-05	1.49E-03	ko05170
Infectious diseases	Tuberculosis	19	5.83E-06	2.03E-04	ko05152
Global and overview maps	Microbial metabolism in diverse environments	19	0.5429525	8.35E-01	ko01120
Energy metabolism	Photosynthesis	19	1	1.00E+00	ko00195
Nervous system	Retrograde endocannabinoid signaling	17	2.25E-05	6.10E-04	ko04723
Infectious diseases	Pathogenic Escherichia coli infection	16	0.000493285	6.02E-03	ko05130
Infectious diseases	Chagas disease (American trypanosomiasis)	15	1.89E-05	5.76E-04	ko05142
Signal transduction	MAPK signaling pathway	15	0.000144833	2.69E-03	ko04010
Infectious disease: bacterial	Yersinia infection	15	0.000309409	4.44E-03	ko05135
Global and overview maps	Carbon metabolism	15	0.7440411	9.16E-01	ko01200
Infectious diseases	Measles	14	0.000309258	4.44E-03	ko05162
Transport and catabolism	Endocytosis	14	0.002767204	2.33E-02	ko04144
Infectious diseases	Leishmaniasis	13	0.000122287	2.49E-03	ko05140
Signal transduction	NF-kappa B signaling pathway	13	0.000154427	2.69E-03	ko04064
Infectious diseases	Herpes simplex infection	13	0.000596411	6.93E-03	ko05168
Infectious diseases	Toxoplasmosis	13	0.001553754	1.57E-02	ko05145
Global and overview maps	Biosynthesis of amino acids	13	0.1040231	3.82E-01	ko01230

Table S21 KEGG pathway enrichment for 2,585 candidate genes were shared by the π ratio and Fst (top30).

KEGG_B_class	Pathway	out	Pvalue	Qvalue	Pathway
Global and overview maps	Metabolic pathways	273	0.8430584	8.89E-01	ko01100
Global and overview maps	Biosynthesis of secondary metabolites	120	5.24E-07	2.86E-05	ko01110
Energy metabolism	Oxidative phosphorylation	52	0.9999997	1.00E+00	ko00190
Environmental adaptation	Thermogenesis	43	2.19E-09	3.58E-07	ko04714
Neurodegenerative diseases	Alzheimer disease	41	6.76E-08	5.53E-06	ko05010
Neurodegenerative diseases	Huntington disease	39	3.58E-07	2.34E-05	ko05016
Translation	Ribosome	37	1	1.00E+00	ko03010
Global and overview maps	Carbon metabolism	36	0.01729145	6.81E-02	ko01200
Neurodegenerative diseases	Parkinson disease	35	1.48E-08	1.62E-06	ko05012
Global and overview maps	Microbial metabolism in diverse environments	30	0.3588238	4.80E-01	ko01120
Signal transduction	Plant hormone signal transduction	28	0.00419201	2.61E-02	ko04075
Endocrine and metabolic diseases	Non-alcoholic fatty liver disease (NAFLD)	26	1.16E-10	3.80E-08	ko04932
Folding, sorting and degradation	Protein processing in endoplasmic reticulum	26	5.90E-05	1.48E-03	ko04141
Global and overview maps	Biosynthesis of amino acids	24	0.002213855	2.07E-02	ko01230
Infectious diseases	Salmonella infection	23	0.008807791	4.30E-02	ko05132
Carbohydrate metabolism	Pentose and glucuronate interconversions	21	3.99E-05	1.19E-03	ko00040
Infectious disease: viral	Human immunodeficiency virus 1 infection	21	0.003051237	2.17E-02	ko05170
Nervous system	Retrograde endocannabinoid signaling	20	8.62E-05	2.01E-03	ko04723
Translation	RNA transport	19	0.002834881	2.16E-02	ko03013
Energy metabolism	Photosynthesis	19	1	1.00E+00	ko00195
Folding, sorting and degradation	Ubiquitin mediated proteolysis	18	0.000152823	3.12E-03	ko04120
Signal transduction	Two-component system	16	2.49E-06	1.02E-04	ko02020
Signal transduction	AMPK signaling pathway	16	1.03E-05	3.38E-04	ko04152
Infectious diseases	Shigellosis	16	0.000207551	3.99E-03	ko05131
Infectious diseases	Human papillomavirus infection	16	0.000390491	6.08E-03	ko05165
Infectious diseases	Tuberculosis	16	0.01155154	5.17E-02	ko05152
Translation	mRNA surveillance pathway	15	0.002916711	2.16E-02	ko03015
Cell growth and death	Cell cycle	15	0.003837944	2.56E-02	ko04110
Infectious diseases	Pathogenic Escherichia coli infection	15	0.03819695	1.24E-01	ko05130
