# Supplementary material for: Chromosome-scale assembly and population diversity analyses provide insights into the evolution of Sapindus mukorossi
Source: Hortic Res. 2022 Feb 17;9:uhac012. doi: 10.1093/hr/uhac012 (PMC8854635; doi:10.1093/hr/uhac012)
Supplement: Web_Material_uhac012 [file web_material_uhac012.zip › supplementary figures.doc]

**Supplementary Figures**

Fig. S1 21-K-mer count distribution for the *S. mukorossi* genome size estimation.

Fig. S2 The size estimation of the *S. mukorossi* genome by flow cytometry. The genome size (1C) of *S. mukorossi* (~380 Mb) was estimated to be approximately 0.43-fold of tomato B73 (~880 Mb) as internal reference.

A	B

Fig. S3 Molecular karyotype analysis of *S. mukorossi*. **a** Karyotyping of *S. mukorossi* chromosomes based on 5S rDNA FISH signals (red). **b** Karyotyping of *S. mukorossi* chromosomes based on 5S rDNA FISH signals (green).

Fig. S4 *S. mukorossi* genome-wide all-by-all Hi-C interactions heat map. The map shows a high resolution of individual chromosomes that are scaffolded and assembled independently.

Fig. S5 Venn diagram of the number of annotated genes in NR, Swiss-Prot, GO, KOG, and KEGG, respectively. The numbers of genes (clusters) are indicated for each database and database intersection.

Fig. S6 Gene flow events. Arrow represents the migration trajectory. Red indicates strong gene flow, yellow indicates weak gene flow.

Fig. S7 Level of Tajima’s D in each subpopulation of *S. mukorossi.*

A

B

Fig. S8 GO term (A) and KEGG pathway (B) enrichment for sweep regions based on the results of Fst.

A

B

Fig. S9 GO term (A) and KEGG pathway (B) enrichment for sweep regions based on the results of π ratio (π_GroupIII_/π_GroupI_).

Fig. S10 KEGG pathway enrichment for 2,585 candidate genes were shared by the π ratio and Fst.

Fig. S11 Manhattan plots of CLR among the Group I and Group III of *S. mukorossi* populations. Red and blue dashed lines indicate the threshold by the top 1% and top 5% of CLR value, respectively. Genes located within the significant CLR peaks and corresponding annotations are denoted.

Fig. S12 Quantile-quantile plot for oil content GWAS under a mixed linear model.

Fig. S13 Quantile-quantile plot for saponins content GWAS under a mixed linear model.

Fig. S14 Quantile-quantile plot for fruit weight GWAS under a mixed linear model.

Fig. S15 Quantile-quantile plot for peel-to-fruit ratio GWAS under a mixed linear model.

Fig. S16 Quantile-quantile plot for seed-to-fruit ratio GWAS under a mixed linear model.

Fig. S17 Quantile-quantile plot for kernel-to-fruit ratio GWAS under a mixed linear model.
