# Supplementary material for: Chromosome-scale assembly and population diversity analyses provide insights into the evolution of Sapindus mukorossi
Source: Hortic Res. 2022 Feb 17;9:uhac012. doi: 10.1093/hr/uhac012 (PMC8854635; doi:10.1093/hr/uhac012)
Supplement: Web_Material_uhac012 [file web_material_uhac012.zip › Supplementary Tables.doc]

**Supplementary Tables**

**Table S1 Summary of sequenced *S. mukorossi*.**

| **Sample Acc.** | **Location** | **Subpopulation** | **Longitude** | **Latitude** | **Altitude**  **/m** | **Age**  **/year** | **Height**  **/m** | **Diameter**  **/cm** | **Crown width/m** | **Canopy transmittance** |
| --- | --- | --- | --- | --- | --- | --- | --- | --- | --- | --- |
| 71 | Anhui province, China | Group I | 117°16′ | 31°52′ | 9 | 30 | 9 | 34 | 10*10 | 3 |
| 72 | Anhui province, China | Group I | 117°16′ | 31°52′ | 9 | 15 | 6 | 18 | 6*5 | 3 |
| 73 | Anhui province, China | Group I | 117°17′ | 31°52′ | 7 | 50 | 13 | 39 | 7*6 | 3 |
| 74 | Anhui province, China | Group I | 117°12′ | 31°52′ | 25 | 20 | 6 | 24 | 8*7 | 4 |
| 75 | Anhui province, China | Group I | 116°30′ | 31°44′ | 50 | 12 | 5 | 14 | 3*3 | 3 |
| 69 | Anhui province, China | Group I | 118°24′ | 31°29′ | 24 | 50 | 12 | 45 | 12*8 | 4 |
| 70 | Anhui province, China | Group I | 118°22′ | 31°19′ | 25 | 20 | 6 | 19 | 6*5 | 3 |
| 28 | Anhui province, China | Group I | 118°38′ | 31°14′ | 9 | 20 | 7 | 18 | 6*6 | 4 |
| 29 | Anhui province, China | Group I | 118°48′ | 31°16′ | 10 | 35 | 8 | 31 | 6*6 | 4 |
| 11 | Fujian province, China | Group II | 118°08′ | 27°02′ | 229 | 50 | 19.5 | 34 | 17.6 | 3 |
| 63 | Fujian province, China | Group II | 118°09′ | 26°59′ | 194 | 100 | 14 | 61 | 10*8 | 3 |
| 8 | Fujian province, China | Group II | 118°07′ | 26°33′ | 79 | 30 | 12 | 46 | 11*12 | 2 |
| 9 | Fujian province, China | Group I | 118°06′ | 26°33′ | 79 | 30 | 12 | 31 | 8*9 | 3 |
| 10 | Fujian province, China | Group I | 118°05′ | 26°34′ | 80 | 50 | 7.5 | 37 | 5*6 | 2 |
| 12 | Fujian province, China | Group I | 118°06′ | 26°33′ | 79 | 20 | 10 | 26 | 8*7 | 3 |
| 66 | Fujian province, China | Group I | 118°30′ | 27°55′ | 248 | 50 | 6 | 31 | 5*6 | 3 |
| 64 | Fujian province, China | Group I | 118°22′ | 27°42′ | 211 | 25 | 11 | 21/25 | 10*10 | 2 |
| 65 | Fujian province, China | Group I | 118°23′ | 27°44′ | 251 | 60 | 11 | 52 | 12*13 | 3 |
| 13 | Fujian province, China | Group I | 117°32′ | 26°11′ | 147 | 41 | 10 | 38 | 8*7 | 3 |
| 14 | Fujian province, China | Group I | 117°37′ | 26°15′ | 174 | 30 | 8 | 38 | 5*5 | 5 |
| 4 | Fujian province, China | Group I | 117°33′ | 26°11′ | 88 | 50 | 13 | 41 | 14*10 | 4 |
| 5 | Fujian province, China | Group I | 117°33′ | 26°11′ | 148 | 15 | 5 | 14 | 5*4 | 3 |
| 22 | Fujian province, China | Group I | 116°42′ | 26°16′ | 466 | 35 | 9 | 29 | 7*6 | 2 |
| 25 | Fujian province, China | Group I | 116°42′ | 26°16 | 374 | 31 | 7.5 | 25 | 3*4 | 3 |
| 27 | Fujian province, China | Group I | 116°45′ | 26°15′ | 368 | 31 | 6.2 | 27 | 7*7 | 5 |
| 21 | Fujian province, China | Group I | 116°48′ | 26°10′ | 344 | 41 | 9 | 35 | 9*9 | 1 |
| 23 | Fujian province, China | Group I | 116°48′ | 26°10′ | 344 | 31 | 9 | 40 | 9*9 | 3 |
| 24 | Fujian province, China | Group I | 116°48′ | 26°10′ | 344 | 41 | 13 | 55 | 9*9 | 2 |
| 6 | Fujian province, China | Group II | 117°50′ | 26°55′ | 248 | 31 | 8 | 28.7 | 7*8 | 3 |
| 7 | Fujian province, China | Group I | 117°52′ | 26°27′ | 110 | 53 | 15 | 38.7 | 8*11 | 4 |
| 1 | Fujian province, China | Group I | 117°54′ | 26°47′ | 110 | 36 | 8.4 | 56 | 7*9 | 3 |
| 2 | Fujian province, China | Group I | 117°54′ | 26°48′ | 110 | 36 | 7 | 40 | 6*10 | 3 |
| 3 | Fujian province, China | Group I | 117°53′ | 26°57′ | 130 | 46 | 8.2 | 43 | 9*8 | 2 |
| 40 | Fujian province, China | Group I | 117°21′ | 25° 59′ | 136 | 31 | 15.5 | 28 | 12*11 | 4 |
| 100 | Guangdong province, China | Group II | 114°12′ | 25°03′ | 150 | 100 | 8 | 42 | 8*8 | 2 |
| 101 | Guangdong province, China | Group II | 114°12′ | 25°02′ | 120 | 100 | 5 | 33 | 5*2 | 4 |
| 106 | Guangxi province, China | Group II | 110°17′ | 25°16′ | 156 | 100 | 12 | 40 | 10*11 | 3 |
| 107 | Guangxi province, China | Group II | 110°18′ | 25°16′ | 200 | 80 | 8 | 31 | 11*8 | 4 |
| 108 | Guangxi province, China | Group II | 109°24′ | 24°16′ | 89 | 100 | 8 | 39 | 6*7 | 3 |
| 109 | Guangxi province, China | Group II | 109°24′ | 24°16′ | 105 | 100 | 11 | 31/37 | 10*9 | 2 |
| 110 | Guangxi province, China | Group II | 108°37′ | 21°56′ | -17 | 40 | 7 | 21 | 6*7 | 3 |
| 111 | Guangxi province, China | Group II | 109°24′ | 22°08′ | 59 | 100 | 10 | 37 | 7*8 | 3 |
| 112 | Guangxi province, China | Group II | 109°35′ | 22°23′ | 198 | 50 | 9 | 23 | 8*9 | 3 |
| 103 | Guangxi province, China | Group II | 111°20′ | 23°28′ | 43 | 200 | 13 | 52 | 15*16 | 3 |
| 104 | Guangxi province, China | Group II | 111°08′ | 23°33′ | 35 | 60 | 13 | 31 | 8*8 | 2 |
| 76 | Hubei province, China | Group II | 114°56′ | 30°12′ | 433 | 100 | 16 | 28 | 5*11 | 2 |
| 77 | Hubei province, China | Group II | 114°56′ | 30°12′ | 426 | 100 | 12 | 45 | 9*7 | 3 |
| 78 | Hubei province, China | Group II | 114°56′ | 30°12′ | 383 | 100 | 14 | 44 | 8*10 | 3 |
| 81 | Hunan province, China | Group II | 112°57′ | 28°10′ | 39 | 60 | 8 | 34 | 10*8 | 3 |
| 82 | Hunan province, China | Group II | 112°57′ | 28°10′ | 39 | 100 | 10 | 68 | 6*9 | 4 |
| 83 | Hunan province, China | Group II | 112°59′ | 28°12′ | 44 | 100 | 18 | 34 | 10*12 | 3 |
| 84 | Hunan province, China | Group II | 112°59′ | 28°12′ | 44 | 100 | 18 | 37 | 10*9 | 3 |
| 85 | Hunan province, China | Group II | 113°29′ | 27°39′ | 63 | 60 | 8 | 23 | 6*12 | 3 |
| 86 | Hunan province, China | Group II | 113°29′ | 27°39′ | 65 | 50 | 8 | 22 | 7*9 | 3 |
| 97 | Jiangxi province, China | Group II | 114°39′ | 25°32′ | 138 | 100 | 12 | 37 | 6*9 | 3 |
| 99 | Jiangxi province, China | Group II | 114°32′ | 25°27′ | 157 | 260 | 10 | 47 | 8*6 | 3 |
| 98 | Jiangxi province, China | Group II | 114°34′ | 25°32′ | 150 | 300 | 11 | 21/25 | 11*10 | 3 |
| 96 | Jiangxi province, China | Group II | 114°45′ | 25°39′ | 136 | 100 | 12 | 57 | 8*7 | 3 |
| 36 | Jiangxi province, China | Group II | 116°10′ | 28°20′ | 50 | 52 | 12 | 56 | 10*11 | 3 |
| 39 | Jiangxi province, China | Group II | 116°08′ | 28° 21′ | 50 | 45 | 13.5 | 36 | 10*12 | 2 |
| 37 | Jiangxi province, China | Group I | 115°46′ | 29°12′ | 55 | 6 | 7 | 12 | 10*12 | 2 |
| 38 | Jiangxi province, China | Group I | 115°48′ | 29°26′ | 837 | 21 | 9 | 23 | 4*4 | 4 |
| 90 | Jiangxi province, China | Group I | 115°54′ | 28°41′ | 62 | 70 | 11 | 23 | 4*5 | 3 |
| 91 | Jiangxi province, China | Group I | 116°00′ | 25°58′ | 236 | 450 | 20 | 64 | 12*14 | 3 |
| 92 | Jiangxi province, China | Group II | 116°00′ | 25°57′ | 236 | 300 | 13 | 55 | 3*4 | 4 |
| 15 | Jiangxi province, China | Group II | 114°34′ | 26°22′ | 106 | 80 | 12 | 41 | 11*13 | 3 |
| 16 | Jiangxi province, China | Group II | 114°03′ | 26°20′ | 130 | 53 | 14 | 55 | 9*8 | 3 |
| 93 | Jiangxi province, China | Group II | 114°53′ | 26°40′ | 126 | 51 | 12.5 | 32/38 | 10*9 | 2 |
| 94 | Jiangxi province, China | Group II | 114°34′ | 26°22′ | 105 | 100 | 13 | 33 | 10*8 | 2 |
| 95 | Jiangxi province, China | Group II | 114°31′ | 26°20′ | 125 | 10 | 6 | 16 | 6*7 | 3 |
| 88 | Jiangxi province, China | Group I | 114°55 | 27°48′ | 69 | 40 | 5 | 21 | 4*4 | 3 |
| 89 | Jiangxi province, China | Group II | 114°54′ | 27°49′ | 65 | 50 | 8 | 30 | 6*8 | 3 |
| 87 | Jiangxi province, China | Group II | 114°23′ | 27°49′ | 98 | 50 | 6 | 23 | 3*3 | 2 |
| 26 | Jiangxi province, China | Group II | 117°01′ | 28°14′ | 148 | 30 | 8 | 16 | 4*3 | 3 |
| 44 | Yunnan province, China | Group III | 102°53′ | 23°44′ | 1384 | >100 | 15.8 | 78 | 12*10 | 3 |
| 46 | Yunnan province, China | Group III | 102°53′ | 23°44′ | 1384 | >100 | 12 | 48 | 11*10 | 3 |
| 113 | Yunnan province, China | Group III | 102°54′ | 23°43′ | 1384 | 40 | 7 | 32 | 3*5 | 2 |
| 116 | Yunnan province, China | Group III | 102°54′ | 23°43′ | 1384 | 30 | 15 | 28 | 5*4 | 3 |
| 41 | Yunnan province, China | Group III | 102°44′ | 25°08′ | 1944 | 430 | 15 | 72 | 7*6 | 2 |
| 42 | Yunnan province, China | Group III | 102°44′ | 25°08′ | 1944 | >400 | 18 | 131 | 10*8 | 3 |
| 43 | Yunnan province, China | Group III | 102°44′ | 25°08′ | 1944 | >400 | 18 | 73 | 6*8 | 3 |
| 56 | Yunnan province, China | Group III | 101°02′ | 23°04′ | 1320 | >80 | 14 | 56 | 9*8 | 3 |
| 57 | Yunnan province, China | Group III | 101°02′ | 23°03′ | 1365 | >80 | 16 | 48 | 10*12 | 3 |
| 48 | Yunnan province, China | Group III | 102°38′ | 23°38′ | 1469 | >45 | 14 | 36 | 9*7 | 3 |
| 49 | Yunnan province, China | Group III | 102°38′ | 23°38′ | 1469 | >45 | 12.5 | 36 | 8*5 | 2 |
| 50 | Yunnan province, China | Group III | 102°22′ | 23°27′ | 1693 | >260 | 14 | 89 | 16*15 | 3 |
| 51 | Yunnan province, China | Group III | 102°22′ | 23°27′ | 1671 | >400 | 13 | 101 | 16*15 | 3 |
| 54 | Yunnan province, China | Group III | 102°22′ | 23°27′ | 1565 | >300 | 17 | 69 | 10*8 | 2 |
| 117 | Yunnan province, China | Group III | 102°22′ | 23°27′ | 1693 | 50 | 15 | 52 | 8*12 | 3 |
| 59 | Yunnan province, China | Group III | 101°53′ | 22°05′ | 749 | >50 | 13 | 52 | 6*8 | 3 |
| 62 | Yunnan province, China | Group III | 101°15′ | 21°55′ | 606 | >35 | 17 | 31 | 6*6 | 3 |
| 60 | Fujian province, China | Group I | 101°55′ | 21°55′ | 546 | >45 | 18 | 45 | 15*14 | 3 |
| 61 | Yunnan province, China | Group III | 101°55′ | 21°55′ | 546 | >50 | 18 | 30.28 | 14*13 | 3 |
| 30 | Zhejiang province, China | Group I | 120°07′ | 30°15′ | 10 | 36 | 11 | 27 | 8*7 | 3 |
| 31 | Zhejiang province, China | Group I | 120°07′ | 30°15′ | 10 | 35 | 10 | 22 | 8*7 | 3 |
| 32 | Zhejiang province, China | Group I | 120°07′ | 30°15′ | 10 | 45 | 10 | 35 | 7*8 | 5 |
| 33 | Zhejiang province, China | Group I | 120°07′ | 30°15′ | 10 | 36 | 9 | 25 | 6*8 | 4 |
| 34 | Zhejiang province, China | Group I | 120°07′ | 30°15′ | 10 | 31 | 11 | 30 | 7*9 | 3 |
| 35 | Zhejiang province, China | Group I | 120°07′ | 30°15′ | 10 | 45 | 10 | 43 | 8*9 | 4 |
| 19 | Zhejiang province, China | Group I | 119°39′ | 29°06′ | 46 | 35 | 13.5 | 36 | 9*16 | 4 |
| 20 | Zhejiang province, China | Group I | 119°39′ | 29°06′ | 25 | 30 | 10 | 28 | 7*8 | 3 |
| 68 | Zhejiang province, China | Group I | 119°35′ | 29°09′ | 73 | 300 | 5.5 | 33 | 4*5 | 4 |
| 17 | Zhejiang province, China | Group I | 118°52′ | 28°57′ | 94 | 108 | 17.9 | 64 | 10*10 | 4 |
| 18 | Zhejiang province, China | Group I | 118°52′ | 28°57′ | 95 | 28 | 9 | 30 | 8*9 | 3 |

**Table S2 Summary of sequenced 57 *S. mukorossi* for GWAS.**

| Sample Acc. | fruit weight/g | peel-to-  fruit ratio | saponins content/% | seed-to-  fruit ratio | kernel-to-  fruit ratio | oil content/% |
| --- | --- | --- | --- | --- | --- | --- |
| 1 | 3.04 | 0.53 | 73.14 | 0.47 | 0.3104 | 43.93 |
| 2 | 1.88 | 0.46 | 80.82 | 0.54 | 0.2993 | 40.74 |
| 3 | 3.03 | 0.63 | 65.79 | 0.37 | 0.2472 | 38.82 |
| 5 | 3.44 | 0.55 | 59.98 | 0.45 | 0.3183 | 38.65 |
| 6 | 1.92 | 0.53 | 81.64 | 0.47 | 0.3261 | 38.96 |
| 8 | 3.63 | 0.67 | 74.95 | 0.33 | 0.2561 | 32.95 |
| 13 | 3.41 | 0.58 | 78.18 | 0.42 | 0.3323 | 38.04 |
| 14 | 3.11 | 0.57 | 64.17 | 0.43 | 0.2931 | 39.54 |
| 15 | 3.59 | 0.59 | 44.05 | 0.41 | 0.2418 | 39.55 |
| 16 | 2.16 | 0.54 | 56.59 | 0.46 | 0.2313 | 41.56 |
| 17 | 3.19 | 0.62 | 69.07 | 0.38 | 0.2567 | 40.18 |
| 18 | 2.24 | 0.58 | 59.94 | 0.42 | 0.2166 | 36.84 |
| 19 | 3.71 | 0.61 | 75.83 | 0.39 | 0.2458 | 36.65 |
| 20 | 3.69 | 0.59 | 61.58 | 0.41 | 0.3197 | 41.63 |
| 22 | 3.09 | 0.64 | 41.53 | 0.36 | 0.2541 | 40.93 |
| 24 | 5.34 | 0.65 | 40.75 | 0.35 | 0.3002 | 37.11 |
| 25 | 2.93 | 0.68 | 72.48 | 0.32 | 0.2252 | 44.28 |
| 26 | 4.51 | 0.64 | 54.95 | 0.36 | 0.2627 | 34.44 |
| 27 | 3.34 | 0.67 | 74.98 | 0.33 | 0.2458 | 40.78 |
| 33 | 3.24 | 0.52 | 49.05 | 0.48 | 0.27 | 35.49 |
| 36 | 2.93 | 0.59 | 59.03 | 0.41 | 0.2804 | 35.06 |
| 37 | 2.29 | 0.61 | 43.33 | 0.39 | 0.2342 | 34.71 |
| 40 | 3.11 | 0.57 | 59.94 | 0.43 | 0.2983 | 40.72 |
| 43 | 2.24 | 0.51 | 77.45 | 0.49 | 0.4229 | 40.56 |
| 56 | 6.35 | 0.69 | 31.64 | 0.31 | 0.2786 | 38.70 |
| 63 | 2.71 | 0.54 | 71.6 | 0.46 | 0.2416 | 33.69 |
| 64 | 2.79 | 0.63 | 54.84 | 0.37 | 0.2767 | 34.81 |
| 65 | 2.42 | 0.53 | 73.33 | 0.47 | 0.2483 | 34.07 |
| 66 | 3.28 | 0.61 | 49.17 | 0.39 | 0.273 | 38.82 |
| 68 | 3.56 | 0.62 | 48.22 | 0.38 | 0.3159 | 34.74 |
| 70 | 4 | 0.5 | 81.56 | 0.5 | 0.3646 | 33.30 |
| 71 | 3.16 | 0.5 | 56.63 | 0.5 | 0.2508 | 43.06 |
| 72 | 3.91 | 0.59 | 72.4 | 0.41 | 0.2883 | 36.83 |
| 73 | 3.52 | 0.57 | 62.23 | 0.43 | 0.3012 | 40.25 |
| 74 | 3.88 | 0.57 | 63.27 | 0.43 | 0.1662 | 41.73 |
| 75 | 2.03 | 0.49 | 71.62 | 0.51 | 0.2869 | 37.46 |
| 81 | 2.1 | 0.49 | 54.13 | 0.51 | 0.2648 | 35.51 |
| 82 | 4.28 | 0.62 | 64.06 | 0.38 | 0.2133 | 37.39 |
| 85 | 2.68 | 0.51 | 69.1 | 0.49 | 0.1692 | 37.40 |
| 86 | 3.04 | 0.59 | 65.66 | 0.41 | 0.2164 | 44.37 |
| 87 | 2.32 | 0.59 | 63.16 | 0.41 | 0.1718 | 46.08 |
| 89 | 2.12 | 0.51 | 67.43 | 0.49 | 0.1563 | 42.86 |
| 90 | 3.28 | 0.69 | 59.86 | 0.31 | 0.2936 | 32.20 |
| 91 | 2.62 | 0.59 | 57.27 | 0.41 | 0.1468 | 33.46 |
| 93 | 2.37 | 0.54 | 53.28 | 0.46 | 0.1447 | 37.70 |
| 94 | 2.73 | 0.5 | 58.29 | 0.5 | 0.2773 | 39.26 |
| 96 | 3.67 | 0.58 | 61.61 | 0.42 | 0.2773 | 38.20 |
| 97 | 2.95 | 0.61 | 61.54 | 0.39 | 0.2662 | 35.04 |
| 98 | 3.44 | 0.61 | 59.94 | 0.39 | 0.2049 | 58.6 |
| 99 | 3.11 | 0.61 | 57.37 | 0.39 | 0.2116 | 36.87 |
| 106 | 3.01 | 0.64 | 53.26 | 0.36 | 0.1974 | 33.43 |
| 107 | 3.25 | 0.64 | 47.45 | 0.36 | 0.1935 | 33.4 |
| 108 | 4.39 | 0.65 | 80.65 | 0.35 | 0.2093 | 41.22 |
| 110 | 4.15 | 0.64 | 44.15 | 0.36 | 0.2605 | 36.16 |
| 112 | 4.17 | 0.56 | 34.92 | 0.44 | 0.3125 | 38.85 |
| 113 | 3.43 | 0.64 | 35.79 | 0.36 | 0.239 | 33.75 |
| 83 | 3.62 | 0.61 | 63.33 | 0.39 | 0.2066 | 33.40 |

Table S3 Sequencing data used for *S. mukorossi* genome construction. Note that the sequence coverage was calculated using the Kmer -based estimated genome size.

| Library  resource | Sequencing platform | Insert size (bp) | Clean data (Gb) | Sequence coverage (X) | Use of the data |
| --- | --- | --- | --- | --- | --- |
| Genome | Illumina HiSeq X Ten | 250 bp | 21 | 47 | Genome estimation |
| Genome | PacBio HiFi | 20 kb | 22.96 | 57.4 | Genome assembly |
| Hi-C | Illumina HiSeq X Ten | 250 bp | 40.96 | 102 | Chromosome construction |
| mRNA | NovaSeq 6000 | 150 bp | 54 | - | Annotation |
| Requencing data | Illumina HiSeq X Ten | 150 bp | 545.83 | - | Population genetic analysis and GWAS analysis |

Table S4 Assembly statistics for the *S. mukorossi* genome

| Items | Hifiasm | | Hi-C | |
| --- | --- | --- | --- | --- |
|  | Contig_len(Mb) | Contig_number | Scaffold_len(Mb) | Scaffold_number |
| Total | 391.55 | 1112 | 391.57 | 1007 |
| Max | 53.08 | - | 44.80 | - |
| Number>=2 kb | - | 1112 | - | 1007 |
| N50 | 2.88 | - | 24.66 | - |

Table S5 Pseudomolecule length statistics after Hi-C assisted assembly

| GroupID | Length | Scaf Num |
| --- | --- | --- |
| LG01 | 44800840 | 30 |
| LG02 | 35436386 | 19 |
| LG03 | 31624267 | 25 |
| LG04 | 27116659 | 15 |
| LG05 | 25299562 | 9 |
| LG06 | 24845014 | 29 |
| LG07 | 24662827 | 18 |
| LG08 | 21895147 | 8 |
| LG09 | 21881448 | 16 |
| LG10 | 21611680 | 6 |
| LG11 | 21130090 | 12 |
| LG12 | 19383384 | 8 |
| LG13 | 17294447 | 42 |
| LG14 | 15756312 | 5 |
| Total | 352738063 | 242 |
| Orangin Genome | 391552161 |  |
| Loading Rate | 0.900871194528 |  |

Table S6 Assessment of the completeness of the S. mukorossi genome assembly by BUSCO

| Type | Number | Percent (%) |
| --- | --- | --- |
| Complete BUSCOs (C) | 356 | 83.8 |
| Complete and single-copy BUSCOs (S) | 351 | 82.6 |
| Complete and duplicated BUSCOs (D) | 5 | 1.2 |
| Fragmented BUSCOs (F) | 7 | 1.6 |
| Missing BUSCOs (M) | 62 | 14.6 |
| Total BUSCO groups searched | 425 | 100 |

Table S7 Transcriptome reads alignment rate of the *S. mukorossi* genome

| Total_EST_Num: | 659372 |
| --- | --- |
| Total_EST_Len: | 557124883 |
| Bases_Covered_By_Assembly: | 0.940969657506915 |
| Seq_Covered_By_Assembly: | 0.402437470805554 |
| Num90: | 230022 |
| Per90: | 0.348850117991058 |
| Num50: | 250017 |
| Per50: | 0.37917442657559 |

Table S8 Annotation statistics for the *S. mukorossi* genome

| Annotation statistics for the genome | | Number | Percent (%) |
| --- | --- | --- | --- |
|  | Total protein | 31,853 |  |
|  | NR | 27,335 | 85.82 |
|  | Swiss-Prot | 23,766 | 74.61 |
|  | GO | 18,615 | 58.44 |
|  | KOG | 14,452 | 45.37 |
|  | KEGG | 11,025 | 34.61 |
|  | In all database | 6,685 | 20.99 |
|  | In at least one database | 27,509 | 86.36 |

Table S9 Transcription factors

| Number | Class |
| --- | --- |
| 850 | protein kinase family protein |
| 424 | pentatricopeptide (PPR) repeat-containing protein |
| 132 | Disease resistance protein (TIR-NBS-LRR class) |
| 93 | basic helix-loop-helix (bHLH) DNA-binding superfamily protein |
| 53 | Integrase-type DNA-binding superfamily protein |
| 42 | Homeodomain-like superfamily protein |
| 40 | AP2/B3-like transcriptional factor family protein |
| 38 | DNAJ heat shock N-terminal domain-containing protein |
| 62 | myb-like transcription factor family protein |
| 29 | Duplicated homeodomain-like superfamily protein |
| 24 | WD-40 repeat family protein / zfwd4 protein (ZFWD4) |
| 24 | Zinc finger C-x8-C-x5-C-x3-H type family protein |
| 22 | C2H2-like zinc finger protein |
| 21 | Basic-leucine zipper (bZIP) transcription factor family protein |
| 21 | GRAS family transcription factor |
| 19 | C2H2 and C2HC zinc fingers superfamily protein |
| 35 | AGAMOUS-like 62 |
| 27 | NAC-domain protein 101 |
| 16 | B-box type zinc finger protein with CCT domain |
| 16 | K-box region and MADS-box transcription factor family protein |
| 14 | zinc finger WD40 repeat protein 1 |
| 12 | NAC (No Apical Meristem) domain transcriptional regulator superfamily protein |
| 12 | myb-like HTH transcriptional regulator family protein |
| 12 | nucleic acid binding;zinc ion binding;DNA binding |
| 12 | zinc finger (C2H2 type) family protein |
| 11 | AGAMOUS-like 103 |
| 11 | auxin response factor 1 |
| 10 | Calmodulin-binding transcription activator protein with CG-1 and Ankyrin domains |
| 10 | Dof-type zinc finger DNA-binding family protein |
| 8 | Far-red impaired responsive (FAR1) family protein |
| 8 | MADS-box transcription factor family protein |
| 8 | Transcriptional factor B3 family protein / auxin-responsive factor AUX/IAA-related |
| 8 | auxin response factor 9 |
| 8 | bZIP transcription factor family protein |
| 8 | sequence-specific DNA binding transcription factors |

Table S10 Repetitive element annotations in the *S. mukorossi.*

==================================================

number of	length	percentage

elements*	occupied of sequence

--------------------------------------------------

Retroelements	38863	36299360 bp	9.27 %

SINEs: 245	13341 bp	0.00 %

Penelope	181	17011 bp	0.00 %

LINEs: 4163	1402605 bp	0.36 %

CRE/SLACS	13	993 bp	0.00 %

L2/CR1/Rex	526	31990 bp	0.01 %

R1/LOA/Jockey	189	8811 bp	0.00 %

R2/R4/NeSL	56	10121 bp	0.00 %

RTE/Bov-B	60	4235 bp	0.00 %

L1/CIN4	2811	1308016 bp	0.33 %

LTR elements: 34455	34883414 bp	8.91 %

BEL/Pao	101	12966 bp	0.00 %

Ty1/Copia	10514	11218738 bp	2.87 %

Gypsy/DIRS1	22239	23310071 bp	5.95 %

Retroviral	378	18787 bp	0.00 %

DNA transposons	15751	2904112 bp	0.74 %

hobo-Activator	4787	1282053 bp	0.33 %

Tc1-IS630-Pogo	924	129162 bp	0.03 %

En-Spm	0	0 bp	0.00 %

MuDR-IS905	0	0 bp	0.00 %

PiggyBac	30	1643 bp	0.00 %

Tourist/Harbinger 1621	372303 bp	0.10 %

Other (Mirage, 283	16802 bp	0.00 %

P-element, Transib)

Rolling-circles	0	0 bp	0.00 %

Unclassified: 1877	518423 bp	0.13 %

Total interspersed repeats: 39721895 bp	10.14 %

Small RNA: 4717	5339089 bp	1.36 %

Satellites: 370	32260 bp	0.01 %

Simple repeats: 233495	9615880 bp	2.46 %

Low complexity: 47729	2513748 bp	0.64 %

==================================================

Table S11 Statistical analysis of non-coding RNAs in *S. mukorossi.*

| Class | number | totalLen | meanLen |
| --- | --- | --- | --- |
| Cis-reg; | 8 | 315 | 39.375 |
| Cis-reg; riboswitch; | 1 | 134 | 134 |
| Gene; | 4 | 1175 | 293.75 |
| Gene; antisense; | 119 | 22236 | 186.8571429 |
| Gene; miRNA; | 88 | 11427 | 129.8522727 |
| Gene; rRNA; | 3035 | 5755584 | 1896.403295 |
| Gene; ribozyme; | 3 | 761 | 253.6666667 |
| Gene; sRNA; | 1 | 389 | 389 |
| Gene; snRNA; snoRNA; CD-box; | 464 | 48196 | 103.8706897 |
| Gene; snRNA; snoRNA; HACA-box; | 29 | 3563 | 122.862069 |
| Gene; snRNA; splicing; | 79 | 11394 | 144.2278481 |
| Gene; tRNA; | 4018 | 303873 | 75.62792434 |
| Intron; | 2675 | 281335 | 105.1719626 |

Table S12 KEGG enrichment of expanded genes.

| KEGG_B_class | Pathway | out |
| --- | --- | --- |
| Energy metabolism | Oxidative phosphorylation | 577 |
| Energy metabolism | Photosynthesis | 541 |
| Transcription | RNA polymerase | 177 |
| Global and overview maps | Metabolic pathways | 1027 |
| Translation | Ribosome | 588 |
| Energy metabolism | Carbon fixation pathways in prokaryotes | 41 |
| Carbohydrate metabolism | Propanoate metabolism | 41 |
| Lipid metabolism | Fatty acid biosynthesis | 41 |
| Cell growth and death | Cell cycle - Caulobacter | 54 |
| Nervous system | Retrograde endocannabinoid signaling | 44 |
| Neurodegenerative diseases | Parkinson disease | 61 |
| Carbohydrate metabolism | Pyruvate metabolism | 41 |
| Global and overview maps | Fatty acid metabolism | 41 |
| Energy metabolism | Carbon fixation in photosynthetic organisms | 52 |
| Carbohydrate metabolism | Glyoxylate and dicarboxylate metabolism | 52 |
| Global and overview maps | Carbon metabolism | 93 |
| Neurodegenerative diseases | Huntington disease | 64 |
| Neurodegenerative diseases | Alzheimer disease | 64 |
| Environmental adaptation | Thermogenesis | 61 |
| Aging | Longevity regulating pathway - worm | 54 |
| Endocrine and metabolic diseases | Non-alcoholic fatty liver disease (NAFLD) | 24 |
| Global and overview maps | Microbial metabolism in diverse environments | 93 |
| Cellular community - eukaryotes | Gap junction | 3 |
| Circulatory system | Cardiac muscle contraction | 3 |
| Metabolism of terpenoids and polyketides | Zeatin biosynthesis | 2 |
| Transcription | Basal transcription factors | 2 |
| Replication and repair | Nucleotide excision repair | 2 |
| Amino acid metabolism | Histidine metabolism | 1 |
| Signal transduction | Ras signaling pathway | 3 |
| Transport and catabolism | Phagosome | 3 |
| Endocrine system | Prolactin signaling pathway | 1 |
| Endocrine system | Ovarian Steroidogenesis | 1 |
| Signal transduction | Two-component system | 3 |
| Lipid metabolism | Steroid hormone biosynthesis | 1 |
| Endocrine and metabolic diseases | Cushing syndrome | 1 |
| Global and overview maps | Biosynthesis of secondary metabolites | 96 |
| Global and overview maps | Biosynthesis of amino acids | 1 |
| Signal transduction | Plant hormone signal transduction | 1 |
| Environmental adaptation | Plant-pathogen interaction | 2 |
| Infectious diseases | Pathogenic Escherichia coli infection | 3 |

Table S13 GO enrichment of expanded genes.

| GO ID (level1) | GO Term (level1) | GO Term (level2) | number_of_out |
| --- | --- | --- | --- |
| GO:0005575 | Cellular Component | organelle | 30 |
| GO:0005575 | Cellular Component | cell | 30 |
| GO:0005575 | Cellular Component | cell part | 30 |
| GO:0005575 | Cellular Component | organelle part | 18 |
| GO:0005575 | Cellular Component | membrane | 14 |
| GO:0008150 | Biological Process | metabolic process | 3 |
| GO:0008150 | Biological Process | cellular process | 3 |
| GO:0005575 | Cellular Component | synapse part | 2 |
| GO:0005575 | Cellular Component | synapse | 2 |
| GO:0003674 | Molecular Function | binding | 1 |
| GO:0003674 | Molecular Function | structural molecule activity | 1 |
| GO:0005575 | Cellular Component | membrane-enclosed lumen | 1 |
| GO:0005575 | Cellular Component | macromolecular complex | 1 |

Table S14 KEGG enrichment of contracted genes (Top 30).

| KEGG_B_class | Pathway | out |
| --- | --- | --- |
| Global and overview maps | Metabolic pathways | 83 |
| Global and overview maps | Biosynthesis of secondary metabolites | 52 |
| Environmental adaptation | Plant-pathogen interaction | 21 |
| Global and overview maps | Microbial metabolism in diverse environments | 17 |
| Global and overview maps | Carbon metabolism | 16 |
| Translation | Ribosome | 12 |
| Transcription | Spliceosome | 11 |
| Biosynthesis of other secondary metabolites | Phenylpropanoid biosynthesis | 11 |
| Global and overview maps | Biosynthesis of amino acids | 11 |
| Nucleotide metabolism | Purine metabolism | 8 |
| Carbohydrate metabolism | Starch and sucrose metabolism | 8 |
| Signal transduction | Plant hormone signal transduction | 8 |
| Infectious diseases | Salmonella infection | 7 |
| Carbohydrate metabolism | Glycolysis / Gluconeogenesis | 6 |
| Infectious diseases | Pathogenic Escherichia coli infection | 6 |
| Infectious disease: viral | Human immunodeficiency virus 1 infection | 6 |
| Folding, sorting and degradation | Protein processing in endoplasmic reticulum | 6 |
| Carbohydrate metabolism | Glyoxylate and dicarboxylate metabolism | 6 |
| Environmental adaptation | Thermogenesis | 6 |
| Endocrine system | Glucagon signaling pathway | 5 |
| Signal transduction | FoxO signaling pathway | 5 |
| Transport and catabolism | Peroxisome | 5 |
| Signal transduction | AMPK signaling pathway | 5 |
| Cell growth and death | Cellular senescence | 5 |
| Immune system | Toll and Imd signaling pathway | 5 |
| Signal transduction | MAPK signaling pathway - plant | 5 |
| Cell growth and death | Oocyte meiosis | 5 |
| Infectious diseases | Herpes simplex infection | 5 |
| Folding, sorting and degradation | Ubiquitin mediated proteolysis | 5 |

Table S15 KEGG enrichment of specific gene (Top 30).

| KEGG_B_class | Pathway | out |
| --- | --- | --- |
| Global and overview maps | Metabolic pathways | 1541 |
| Translation | Ribosome | 1467 |
| Energy metabolism | Photosynthesis | 971 |
| Energy metabolism | Oxidative phosphorylation | 687 |
| Global and overview maps | Biosynthesis of secondary metabolites | 179 |
| Transcription | RNA polymerase | 121 |
| Global and overview maps | Microbial metabolism in diverse environments | 119 |
| Global and overview maps | Carbon metabolism | 116 |
| Energy metabolism | Carbon fixation in photosynthetic organisms | 92 |
| Carbohydrate metabolism | Glyoxylate and dicarboxylate metabolism | 88 |
| Aging | Longevity regulating pathway - worm | 87 |
| Cell growth and death | Cell cycle - Caulobacter | 81 |
| Global and overview maps | Biosynthesis of amino acids | 30 |
| Infectious diseases | Salmonella infection | 25 |
| Environmental adaptation | Plant-pathogen interaction | 24 |
| Translation | RNA transport | 21 |
| Infectious diseases | Shigellosis | 17 |
| Cell growth and death | Cell cycle | 17 |
| Infectious disease: viral | Human immunodeficiency virus 1 infection | 17 |
| Global and overview maps | Fatty acid metabolism | 16 |
| Neurodegenerative diseases | Huntington disease | 16 |
| Lipid metabolism | Fatty acid biosynthesis | 15 |
| Folding, sorting and degradation | Protein processing in endoplasmic reticulum | 15 |
| Neurodegenerative diseases | Alzheimer disease | 15 |
| Infectious diseases | Pathogenic Escherichia coli infection | 15 |
| Cancers | Pathways in cancer | 15 |
| Carbohydrate metabolism | Pyruvate metabolism | 14 |
| Biosynthesis of other secondary metabolites | Phenylpropanoid biosynthesis | 14 |
| Cell growth and death | Cell cycle - yeast | 14 |

Table S16 KEGG enrichment of positively selected genes.

| ID | KO | Kegg | Pathway | Class |
| --- | --- | --- | --- | --- |
| whz_000032 | K03883 | ko00190 | Oxidative phosphorylation | Metabolism |
| whz_000032 | K03883 | ko04723 | Retrograde endocannabinoid signaling | Organismal Systems |
| whz_000032 | K03883 | ko04714 | Thermogenesis | Organismal Systems |
| whz_000032 | K03883 | ko05012 | Parkinson disease | Human Diseases |
| whz_000208 | K14491 | ko04075 | Plant hormone signal transduction | Environmental Information Processing |
| whz_000548 | K14508 | ko04075 | Plant hormone signal transduction | Environmental Information Processing |
| whz_001817 | K01183 | ko00520 | Amino sugar and nucleotide sugar metabolism | Metabolism |
| whz_003329 | K13459 | ko04626 | Plant-pathogen interaction | Organismal Systems |
| whz_004052 | K03361 | ko04120 | Ubiquitin mediated proteolysis | Genetic Information Processing |
| whz_004052 | K03361 | ko04111 | Cell cycle - yeast | Cellular Processes |
| whz_004052 | K10260 | ko04120 | Ubiquitin mediated proteolysis | Genetic Information Processing |
| whz_004053 | K03361 | ko04120 | Ubiquitin mediated proteolysis | Genetic Information Processing |
| whz_004053 | K03361 | ko04111 | Cell cycle - yeast | Cellular Processes |
| whz_004053 | K10260 | ko04120 | Ubiquitin mediated proteolysis | Genetic Information Processing |
| whz_004777 | K02703 | ko00195 | Photosynthesis | Metabolism |
| whz_004777 | K03243 | ko03013 | RNA transport | Genetic Information Processing |
| whz_006074 | K03879 | ko00190 | Oxidative phosphorylation | Metabolism |
| whz_006074 | K03879 | ko04723 | Retrograde endocannabinoid signaling | Organismal Systems |
| whz_006074 | K03879 | ko04714 | Thermogenesis | Organismal Systems |
| whz_006074 | K03879 | ko05012 | Parkinson disease | Human Diseases |
| whz_006095 | K03879 | ko00190 | Oxidative phosphorylation | Metabolism |
| whz_006095 | K03879 | ko04723 | Retrograde endocannabinoid signaling | Organismal Systems |
| whz_006095 | K03879 | ko04714 | Thermogenesis | Organismal Systems |
| whz_006095 | K03879 | ko05012 | Parkinson disease | Human Diseases |

Table S17 GO term enrichment for sweep regions based on the results of Fst.

| GO Term (level1) | GO ID (level2) | GO Term (level2) | number_of_out (All) |
| --- | --- | --- | --- |
| Cellular Component | GO:0005623 | cell | 51 |
| Cellular Component | GO:0044464 | cell part | 51 |
| Cellular Component | GO:0043226 | organelle | 50 |
| Cellular Component | GO:0044422 | organelle part | 32 |
| Cellular Component | GO:0016020 | membrane | 30 |
| Biological Process | GO:0008152 | metabolic process | 6 |
| Biological Process | GO:0009987 | cellular process | 6 |
| Biological Process | GO:0044699 | single-organism process | 4 |
| Biological Process | GO:0050896 | response to stimulus | 3 |
| Molecular Function | GO:0003824 | catalytic activity | 3 |
| Cellular Component | GO:0032991 | macromolecular complex | 3 |
| Biological Process | GO:0051179 | localization | 2 |
| Molecular Function | GO:0005198 | structural molecule activity | 2 |
| Cellular Component | GO:0044425 | membrane part | 2 |
| Cellular Component | GO:0044456 | synapse part | 2 |
| Cellular Component | GO:0045202 | synapse | 2 |
| Biological Process | GO:0032501 | multicellular organismal process | 1 |
| Biological Process | GO:0032502 | developmental process | 1 |
| Biological Process | GO:0040011 | locomotion | 1 |
| Biological Process | GO:0050789 | regulation of biological process | 1 |
| Biological Process | GO:0065007 | biological regulation | 1 |
| Biological Process | GO:0071840 | cellular component organization or biogenesis | 1 |
| Molecular Function | GO:0005215 | transporter activity | 1 |
| Molecular Function | GO:0005488 | binding | 1 |
| Cellular Component | GO:0030054 | cell junction | 1 |
| Cellular Component | GO:0031974 | membrane-enclosed lumen | 1 |
| Cellular Component | GO:0055044 | symplast | 1 |

Table S18 KEGG pathway enrichment for sweep regions based on the results of Fst (Top 30).

| KEGG_B_class | Pathway | out | Pvalue | Qvalue | Pathway |
| --- | --- | --- | --- | --- | --- |
| Global and overview maps | Metabolic pathways | 398 | 0.9937663 | 1.00E+00 | ko01100 |
| Global and overview maps | Biosynthesis of secondary metabolites | 179 | 3.31E-09 | 1.65E-07 | ko01110 |
| Energy metabolism | Oxidative phosphorylation | 83 | 1 | 1.00E+00 | ko00190 |
| Environmental adaptation | Thermogenesis | 73 | 2.00E-18 | 5.52E-16 | ko04714 |
| Neurodegenerative diseases | Huntington disease | 71 | 1.28E-16 | 1.49E-14 | ko05016 |
| Neurodegenerative diseases | Alzheimer disease | 71 | 3.17E-16 | 2.76E-14 | ko05010 |
| Translation | Ribosome | 66 | 1 | 1.00E+00 | ko03010 |
| Neurodegenerative diseases | Parkinson disease | 62 | 3.17E-18 | 5.52E-16 | ko05012 |
| Global and overview maps | Carbon metabolism | 47 | 0.0775807 | 1.43E-01 | ko01200 |
| Signal transduction | Plant hormone signal transduction | 45 | 7.95E-05 | 1.20E-03 | ko04075 |
| Nervous system | Retrograde endocannabinoid signaling | 40 | 1.71E-12 | 1.19E-10 | ko04723 |
| Global and overview maps | Microbial metabolism in diverse environments | 40 | 0.6804587 | 7.49E-01 | ko01120 |
| Infectious diseases | Salmonella infection | 39 | 6.97E-05 | 1.15E-03 | ko05132 |
| Environmental adaptation | Plant-pathogen interaction | 36 | 0.1525421 | 2.37E-01 | ko04626 |
| Endocrine and metabolic diseases | Non-alcoholic fatty liver disease (NAFLD) | 32 | 1.22E-10 | 7.10E-09 | ko04932 |
| Carbohydrate metabolism | Pentose and glucuronate interconversions | 31 | 7.63E-07 | 2.95E-05 | ko00040 |
| Folding, sorting and degradation | Protein processing in endoplasmic reticulum | 31 | 0.000949682 | 6.49E-03 | ko04141 |
| Global and overview maps | Biosynthesis of amino acids | 31 | 0.006641379 | 2.74E-02 | ko01230 |
| Infectious disease: viral | Human immunodeficiency virus 1 infection | 28 | 0.004444804 | 2.18E-02 | ko05170 |
| Infectious diseases | Pathogenic Escherichia coli infection | 27 | 0.000532038 | 4.73E-03 | ko05130 |
| Infectious diseases | Tuberculosis | 26 | 0.000481729 | 4.53E-03 | ko05152 |
| Transport and catabolism | Endocytosis | 25 | 0.00154457 | 9.28E-03 | ko04144 |
| Translation | RNA transport | 25 | 0.004565632 | 2.21E-02 | ko03013 |
| Carbohydrate metabolism | Starch and sucrose metabolism | 24 | 0.00178827 | 1.04E-02 | ko00500 |
| Folding, sorting and degradation | Ubiquitin mediated proteolysis | 23 | 0.000242308 | 2.72E-03 | ko04120 |
| Infectious diseases | Epstein-Barr virus infection | 23 | 0.003496646 | 1.84E-02 | ko05169 |
| Nervous system | Neurotrophin signaling pathway | 22 | 0.00119081 | 7.67E-03 | ko04722 |
| Signal transduction | Two-component system | 21 | 5.21E-07 | 2.26E-05 | ko02020 |
| Infectious diseases | Shigellosis | 21 | 0.000157304 | 2.03E-03 | ko05131 |

Table S19 GO term enrichment for sweep regions based on the results of π ratio (π_GroupIII_/π_GroupI_).

| GO Term (level1) | GO ID (level2) | GO Term (level2) | number_of_out (All) |
| --- | --- | --- | --- |
| Cellular Component | GO:0005623 | cell | 51 |
| Cellular Component | GO:0044464 | cell part | 51 |
| Cellular Component | GO:0043226 | organelle | 50 |
| Cellular Component | GO:0044422 | organelle part | 32 |
| Cellular Component | GO:0016020 | membrane | 30 |
| Biological Process | GO:0008152 | metabolic process | 6 |
| Biological Process | GO:0009987 | cellular process | 6 |
| Biological Process | GO:0044699 | single-organism process | 4 |
| Biological Process | GO:0050896 | response to stimulus | 3 |
| Molecular Function | GO:0003824 | catalytic activity | 3 |
| Cellular Component | GO:0032991 | macromolecular complex | 3 |
| Biological Process | GO:0051179 | localization | 2 |
| Molecular Function | GO:0005198 | structural molecule activity | 2 |
| Cellular Component | GO:0044425 | membrane part | 2 |
| Cellular Component | GO:0044456 | synapse part | 2 |
| Cellular Component | GO:0045202 | synapse | 2 |
| Biological Process | GO:0032501 | multicellular organismal process | 1 |
| Biological Process | GO:0032502 | developmental process | 1 |
| Biological Process | GO:0040011 | locomotion | 1 |
| Biological Process | GO:0050789 | regulation of biological process | 1 |
| Biological Process | GO:0065007 | biological regulation | 1 |
| Biological Process | GO:0071840 | cellular component organization or biogenesis | 1 |
| Molecular Function | GO:0005215 | transporter activity | 1 |
| Molecular Function | GO:0005488 | binding | 1 |
| Cellular Component | GO:0030054 | cell junction | 1 |
| Cellular Component | GO:0031974 | membrane-enclosed lumen | 1 |
| Cellular Component | GO:0055044 | symplast | 1 |

Table S20 KEGG pathway enrichment for sweep regions based on the results of π ratio (π_GroupIII_/π_GroupI_) (Top30)

| KEGG_B_class | Pathway | out | Pvalue | Qvalue | Pathway |
| --- | --- | --- | --- | --- | --- |
| Global and overview maps | Metabolic pathways | 164 | 0.9991305 | 1.00E+00 | ko01100 |
| Global and overview maps | Biosynthesis of secondary metabolites | 65 | 0.05425978 | 2.55E-01 | ko01110 |
| Translation | Ribosome | 64 | 0.9999931 | 1.00E+00 | ko03010 |
| Energy metabolism | Oxidative phosphorylation | 52 | 0.9435328 | 1.00E+00 | ko00190 |
| Neurodegenerative diseases | Alzheimer disease | 38 | 5.36E-11 | 7.02E-09 | ko05010 |
| Environmental adaptation | Thermogenesis | 35 | 1.05E-09 | 8.50E-08 | ko04714 |
| Neurodegenerative diseases | Huntington disease | 35 | 2.00E-09 | 1.22E-07 | ko05016 |
| Neurodegenerative diseases | Parkinson disease | 32 | 5.76E-11 | 7.02E-09 | ko05012 |
| Environmental adaptation | Plant-pathogen interaction | 29 | 9.45E-05 | 2.10E-03 | ko04626 |
| Signal transduction | Plant hormone signal transduction | 25 | 0.000170131 | 2.77E-03 | ko04075 |
| Translation | RNA transport | 22 | 4.75E-07 | 2.32E-05 | ko03013 |
| Infectious diseases | Salmonella infection | 21 | 0.000405034 | 5.20E-03 | ko05132 |
| Infectious disease: viral | Human immunodeficiency virus 1 infection | 20 | 6.11E-05 | 1.49E-03 | ko05170 |
| Infectious diseases | Tuberculosis | 19 | 5.83E-06 | 2.03E-04 | ko05152 |
| Global and overview maps | Microbial metabolism in diverse environments | 19 | 0.5429525 | 8.35E-01 | ko01120 |
| Energy metabolism | Photosynthesis | 19 | 1 | 1.00E+00 | ko00195 |
| Nervous system | Retrograde endocannabinoid signaling | 17 | 2.25E-05 | 6.10E-04 | ko04723 |
| Infectious diseases | Pathogenic Escherichia coli infection | 16 | 0.000493285 | 6.02E-03 | ko05130 |
| Infectious diseases | Chagas disease (American trypanosomiasis) | 15 | 1.89E-05 | 5.76E-04 | ko05142 |
| Signal transduction | MAPK signaling pathway | 15 | 0.000144833 | 2.69E-03 | ko04010 |
| Infectious disease: bacterial | Yersinia infection | 15 | 0.000309409 | 4.44E-03 | ko05135 |
| Global and overview maps | Carbon metabolism | 15 | 0.7440411 | 9.16E-01 | ko01200 |
| Infectious diseases | Measles | 14 | 0.000309258 | 4.44E-03 | ko05162 |
| Transport and catabolism | Endocytosis | 14 | 0.002767204 | 2.33E-02 | ko04144 |
| Infectious diseases | Leishmaniasis | 13 | 0.000122287 | 2.49E-03 | ko05140 |
| Signal transduction | NF-kappa B signaling pathway | 13 | 0.000154427 | 2.69E-03 | ko04064 |
| Infectious diseases | Herpes simplex infection | 13 | 0.000596411 | 6.93E-03 | ko05168 |
| Infectious diseases | Toxoplasmosis | 13 | 0.001553754 | 1.57E-02 | ko05145 |
| Global and overview maps | Biosynthesis of amino acids | 13 | 0.1040231 | 3.82E-01 | ko01230 |

Table S21 KEGG pathway enrichment for 2,585 candidate genes were shared by the π ratio and Fst (top30).

| KEGG_B_class | Pathway | out | Pvalue | Qvalue | Pathway |
| --- | --- | --- | --- | --- | --- |
| Global and overview maps | Metabolic pathways | 273 | 0.8430584 | 8.89E-01 | ko01100 |
| Global and overview maps | Biosynthesis of secondary metabolites | 120 | 5.24E-07 | 2.86E-05 | ko01110 |
| Energy metabolism | Oxidative phosphorylation | 52 | 0.9999997 | 1.00E+00 | ko00190 |
| Environmental adaptation | Thermogenesis | 43 | 2.19E-09 | 3.58E-07 | ko04714 |
| Neurodegenerative diseases | Alzheimer disease | 41 | 6.76E-08 | 5.53E-06 | ko05010 |
| Neurodegenerative diseases | Huntington disease | 39 | 3.58E-07 | 2.34E-05 | ko05016 |
| Translation | Ribosome | 37 | 1 | 1.00E+00 | ko03010 |
| Global and overview maps | Carbon metabolism | 36 | 0.01729145 | 6.81E-02 | ko01200 |
| Neurodegenerative diseases | Parkinson disease | 35 | 1.48E-08 | 1.62E-06 | ko05012 |
| Global and overview maps | Microbial metabolism in diverse environments | 30 | 0.3588238 | 4.80E-01 | ko01120 |
| Signal transduction | Plant hormone signal transduction | 28 | 0.00419201 | 2.61E-02 | ko04075 |
| Endocrine and metabolic diseases | Non-alcoholic fatty liver disease (NAFLD) | 26 | 1.16E-10 | 3.80E-08 | ko04932 |
| Folding, sorting and degradation | Protein processing in endoplasmic reticulum | 26 | 5.90E-05 | 1.48E-03 | ko04141 |
| Global and overview maps | Biosynthesis of amino acids | 24 | 0.002213855 | 2.07E-02 | ko01230 |
| Infectious diseases | Salmonella infection | 23 | 0.008807791 | 4.30E-02 | ko05132 |
| Carbohydrate metabolism | Pentose and glucuronate interconversions | 21 | 3.99E-05 | 1.19E-03 | ko00040 |
| Infectious disease: viral | Human immunodeficiency virus 1 infection | 21 | 0.003051237 | 2.17E-02 | ko05170 |
| Nervous system | Retrograde endocannabinoid signaling | 20 | 8.62E-05 | 2.01E-03 | ko04723 |
| Translation | RNA transport | 19 | 0.002834881 | 2.16E-02 | ko03013 |
| Energy metabolism | Photosynthesis | 19 | 1 | 1.00E+00 | ko00195 |
| Folding, sorting and degradation | Ubiquitin mediated proteolysis | 18 | 0.000152823 | 3.12E-03 | ko04120 |
| Signal transduction | Two-component system | 16 | 2.49E-06 | 1.02E-04 | ko02020 |
| Signal transduction | AMPK signaling pathway | 16 | 1.03E-05 | 3.38E-04 | ko04152 |
| Infectious diseases | Shigellosis | 16 | 0.000207551 | 3.99E-03 | ko05131 |
| Infectious diseases | Human papillomavirus infection | 16 | 0.000390491 | 6.08E-03 | ko05165 |
| Infectious diseases | Tuberculosis | 16 | 0.01155154 | 5.17E-02 | ko05152 |
| Translation | mRNA surveillance pathway | 15 | 0.002916711 | 2.16E-02 | ko03015 |
| Cell growth and death | Cell cycle | 15 | 0.003837944 | 2.56E-02 | ko04110 |
| Infectious diseases | Pathogenic Escherichia coli infection | 15 | 0.03819695 | 1.24E-01 | ko05130 |
